# Diversity and Characterization of Endophytic Fungi Isolated From the Tropical Mangrove Species, *Rhizophora mucronata*, and Identification of Potential Antagonists Against the Soil-Borne Fungus, *Fusarium solani*

**DOI:** 10.3389/fmicb.2018.01707

**Published:** 2018-07-25

**Authors:** Tuan Noraida Tuan Hamzah, Shiou Yih Lee, Asep Hidayat, Razak Terhem, Ibrahim Faridah-Hanum, Rozi Mohamed

**Affiliations:** ^1^Forest Biotech Laboratory, Department of Forest Management, Faculty of Forestry, Universiti Putra Malaysia, Serdang, Malaysia; ^2^Forest Microbiology Laboratory, Forestry and Environment Research, Development and Innovation Agency, Bogor, Indonesia; ^3^Department of Forest Management, Faculty of Forestry, Universiti Putra Malaysia, Serdang, Malaysia

**Keywords:** fungal endophyte, ITS, dual culture, minimum inhibitory concentration, scavenging activity

## Abstract

*Rhizophora mucronata* is an important ecosystem entity of the Malaysian mangrove forest. Since the species grows in a harsh environment, any organism that is isolated from this species would be of huge interest due to its potential in having novel bioactive compounds. In the present work, we isolated, identified and characterized, a total of 78 fungal isolates harboring inside the leaf tissues of *R. mucronata*. Molecular identification using the nuclear ribosomal DNA internal transcribe spacer (ITS) sequences returned with high similarity matches to known sequences in the GenBank. Maximum likelihood analysis revealed the phylogenetic relationship of all isolates from this study. Most of the dominating fungal endophytes were from the genera *Pestalotiopsis*, followed by *Alternaria* and *Cladosporium*. Six isolates representing the genera *Alternaria, Fusarium, Nigrospora, Pestalotiopsis, Phoma*, and *Xylaria*, were further screened for their antagonism activities. Dual culture test assay revealed their inhibition percentages against the phytopathogenic fungus *Fusarium solani* between 45–66%, and 0.8–23% when using non-volatile test assay. Of the six isolates, only *Fusarium lateritium* and *Xylaria* sp. showed antibacterial activities against the pathogenic bacteria, *Bacillus subtilis, Escherichia coli, Pseudomonas aeruginosa*, and *Staphylococcus aureus*, with the Minimum Inhibitory Concentration (MIC) and Minimum Bactericidal Concentration (MBC) ranging from 0.5 to 2 mg/mL. The DPPH radical scavenging assay recorded a high level of antioxidant activity in *Xylaria* sp., 3-fold above that of *F*. *lateritium*. We demonstrate for the first time, two members belonging to the endophytic fungal community in the tropical mangrove species that have potential use as antagonists and antibacterial agents for future biotechnological applications.

## Introduction

Mangroves of South and Southeast Asia comprise 41.4% of the global mangroves (Singh et al., 2014). There are ~70 species of true mangrove species, belonging to 17 different families. The largest mangrove family, Rhizophoraceae, comprises of four mangrove genera: *Bruguiera, Ceriops, Kandelia*, and *Rhizophora* (Tomlinson, 1986; Polidoro et al., 2010). Of these genera, *Rhizophora* has the widest distribution and is commonly found growing in the tropical and subtropical coastal areas (Duke, 2006). Recorded with a total of six true mangrove species and one hybrid names (The Plant List, 2013), three of these species: *R*. *apiculata, R*. *mucronata*, and *R*. *stylosa*, could be found populating the Indo-Malaya region (Duke et al., 2007). *Rhizophora mucronata* (Malay: *bakau kurap*) is characterized by its broadly elliptic to oblong leaves with yellowish stalked flowers (Qin and Boufford, 2007; Setyawan and Ulumuddin, 2012).

Different parts of *R*. *mucronata* such as the bark and leaves have been used traditionally as astringent and antiseptic, and have been shown to possess activities against bacteria, ulcers, and inflammation (Kokpol et al., 1990; Suganthy and Pandima Devi, 2016). Furthermore, pharmacological studies have shown that the leaf exhibits antiviral, anti-inflammatory, and anti-diabetic activities (Gaffar et al., 2011). The leaves also contain (+)-catechin, which has properties in treating Alzheimer disease (Suganthy and Pandima Devi, 2016). Apart from focusing on the plant itself, several studies have been conducted on microorganisms associated with *R*. *mucronata*, including fungal endophytes. Endophytic fungi from the genera *Penicillium, Ampelomyces*, and *Fusarium*, isolated from *R. mucronata* were found to be active against *E. coli* (Prihanto et al., 2011), while several others originating from *R*. *apiculata*, have led to the discovery of novel compounds including acropyrone, bicytosporone D, waol acid, and pestalotiopene (Klaiklay et al., 2012). Fungi from marine environments grow in habitats with unique conditions that attributed to the activation of metabolic pathways and the synthesis of distinct unknown molecules (de Souza Sebastianes et al., 2013). Production of these compounds aids in supporting the adaptation and survival of the fungi in marine ecosystems (Fox and Howlett, 2008).

Endophytic fungi live internally within various tissues of a host plant, asymptomatically, without causing any negative effect to the host plant (Aly et al., 2011). When a host plant harbors endophytes, their concurrence may help the host to adapt to biotic and abiotic stress factors (Hartley et al., 2015; Amin, 2016; Potshangbam et al., 2017). Tolerance to biotic stress as shown by some plants has been associated with fungal natural products (Rodriguez et al., 2008; Douanla-Meli et al., 2013). Despite endophytic fungi being regarded as new sources of novel active compounds, biological activity, and biotechnological developments; their true potential remains underexplored (Debbab et al., 2011).

In general, leaves have a more diverse fungal endophytes community when compared to other parts of the plant (Bayman et al., 1997; Shreelalitha and Sridhar, 2015). For endophytic fungi of mangrove leaf origin, significant biological activities have been reported from these fungi such as having antibiotic, anticancer, antimicrobial and insecticidal properties (Chaeprasert et al., 2010; Abraham et al., 2015; Thomas et al., 2016). The current study was designed to determine species diversity of the endophytic fungal community in the leaf tissue of *R. mucronata*, a tropical mangrove species commonly found at the Matang Mangrove Forest Reserve (MMFR), Perak, Malaysia, and to characterize biological activities of several selected species. These fungal endophytes might offer novel species or strains that possess valuable bioactive compounds, which demonstrates the significance of their study.

## Materials and methods

### Plant materials

Six marine mangrove trees of *R. mucronata* were identified from the Matang Mangrove Forest Reserve (MMFR) (coordinate E 100°3649.1 N 04°5049.1). The trees' diameter breast heights were about 12–14 cm. From each tree, ten mature (dark green) leaves were randomly sampled from the lowest branch, making a total of 60 leaves sampled. The leaves were kept in polyethene bags and brought back directly to the laboratory to be processed immediately. The leaves were washed thoroughly under running tap water to remove adherent debris on the surface of the leaves. Each leaf was cut into five 0.5 cm^2^ segments using a blade, accumulated to 300 leaf segments in total.

### Endophytic fungi isolation and cultivation

Leaf segments were surface-sterilized according to the protocol suggested by Kjer et al. (2010). The segments were washed under running tap water, immersed in 70% ethanol for 60–120 s, followed by soaking in 4% NaOCl for 60 s, rinsed several times in sterile distilled water and then dried on a sterile filter paper. For control, the final sterile water rinse was plated and observed post-incubation period. Absence of fungal growth indiciate the leaf surface was sterile. The sterilized sample was excised 1–2 mm from the edge and the explant plated on a special-made agar medium, Medium A (Kjer et al., 2010). Medium A contained bacto agar (15 g/L), malt extract (15 g/L), artificial sea salt (10 g/L), chloramphenicol (0.2 g/L), and sterile distilled water (1 L), with the final pH adjusted from 7.4 to 7.8. The plates were incubated at 27°C for 7 days. Hyphae tips that grew out from the cultivated leaf segments were sub-cultured into fresh plates containing another special-made agar medium, Medium B (Kjer et al., 2010). Medium B contained similar ingredients to medium A, but without chloramphenicol. Pure cultures were grouped according to colony color, form, elevation and margin characteristics on Medium B. Based on the groupings, isolates with distinct morphology were selected for molecular identification. Cultures were maintained on Medium B for 7 days before being used for genomic DNA extraction and as inoculum for production of fungal extracts. For bioactivity assays, pure cultures were grown on Potato Dextrose Agar (PDA) (Friendemann Schmidt, Australia) at room temperature for 7 days.

### DNA extraction and PCR amplification

Approximately 100 mg of fungal mycelia was used for fungal genomic DNA extraction. Fungal genomic DNA was extracted as previously described by Landum et al. (2016), in accordance to the manufacturer's instructions, using the DNeasy Plant Minikit (Qiagen, Germany). The nuclear ribosomal DNA internal transcribed spacer (ITS) of the fungal isolates were amplified using the forward primer, ITS1-F (5′-CTTGGTCATTTAGAGGAAGTAA-3′ and the reverse primer, ITS4 (5′-CTTGGTCATTTAGAGGAAGTAA-3′) (White et al., 1990). The final reaction volume was 25 μL, containing 12.5 μL of 2X PCRBio Taq Mix Red (PCR Biosystems, UK), 0.4 μM of forward and reverse primers, and 10 ng of genomic DNA template. For negative control, the DNA was replaced with distilled water to verify absence of contamination. PCR was carried out using MyCycler™ (Bio-Rad, USA), programmed for 1 min 95°C; 35 cycles for 15 s at 95°C, 15 s at 55°C, and 1 min at 72°C; and a final 10 min extension at 72°C. The PCR products were separated using 1% agarose gel in 1X TAE buffer (90 mM Tris-acetate and 2 nM EDTA, pH 8.0), stained with ethidium bromide (0.5 μg/mL) and documented using FluorChem™ (Alpha Innotech, USA). PCR products were sent for direct bi-directionally sequencing using ABI PRISM 3730 × 1 Genetic Analyzer (Applied Biosystems, USA) at the First BASE Laboratory Sdn. Bhd., Selangor, Malaysia.

### Sequence and phylogenetic analysis

The resulting DNA sequences were aligned using MUSCLE software embedded in MEGA 7 (Kumar et al., 2016), manually trimmed and edited to obtain complete sequences. Homology searches were carried out using the BLASTn program against the NCBI GenBank database (https://blast.ncbi.nlm.nih.gov/Blast.cgi). DNA substitution models which were suitable for the ITS gene that were assessed using the “find best DNA/Protein Models (ML)” function embedded in the software MEGA 7 by implementing the maximum likelihood (ML) statistical method to test the goodness of fit to several models of evolution. According to the estimated values of all parameters for each model, the model best fitting to the dataset from the ITS sequences was general time reversible (GTR) model and gamma distributed (+G) with invariant sites (+I) (= GTR+G+I) model. ML tree was constructed using MEGA 7 with all positions containing gaps and missing data were included for analysis. Clade supports were calculated based on 1,000 bootstrap re-samplings. A soil fungus, *Mortierella longigemmata* (phyla Zygomycota, family Mortierellaceae) was included as out-group.

### Fungal isolates for antagonism assays

Identified endophytic fungal isolates, which displayed distinct pigmentation and have been previously reported as having some biological activities (Hamzah, 2018) were selected for further analysis. They were *Alternaria macrospora, Fusarium lateritium, Nigrospora oryzae, Pestalotiopsis* sp., *Phoma* sp., and *Xylaria* sp. Fungi were cultivated on PDA plates at 26°C for 7 days. Antagonistic activity of the six fungal isolates were evaluated through the dual culture plate assay (Coşkuntuna and Özer, 2008), and the non-volatile compound test (Hajieghrari et al., 2008), against a known prevalent fungal pathogen, *Fusarium solani*. The phytopathogenic *F*. *solani* strain used in this study was isolated from a tropical tree, *Aquilaria* sp., (Faizal et al., 2017) and maintained at 27°C on PDA media at the Research, Development and Innovation Agency of the Indonesian Ministry of Environment and Forestry (FOERDIA), Indonesia. For all antagonism assays, culture of 7-day-old was used.

### Dual culture plate assay

Fungal isolates were screened using *in vitro* dual culture assays for their ability to suppress the mycelial growth of *F. solani*. A fungal disc (5 mm) was taken from the test pathogen and placed 3 cm from the margin of the PDA plate (8 cm in diameter). A 5 mm disc of the endophytic fungus/antagonist was also placed in a similar manner but on the direct opposite of the pathogen disc. The plate was incubated at room temperature for 7 days. Plates inoculated with *F*. *solani* in the absence of antagonist fungus served as negative controls. The assay was replicated three times. Observations were carried out for 7 days, after which the mycelial radial growth of test pathogen on a control plate (r1) and in the direction of the antagonistic fungus (r2) were measured and the percentage inhibition (*I*%) in mycelial growth was calculated according to the formula (Hajieghrari et al., 2008): *I*% = [(r1–r2)/r1] × 100. Data in the form of *I*% were analyzed statistically with ANOVA using the SAS statistical package (SAS Institute, Cary, NC). For significant effect, the LSD was performed at *P* ≤ 0.05.

Clear inhibition zone (if any) was recorded, and the interactions between *F. solani* and fungal isolates were assessed using a classification system ranged from type A to F: “(1) Type A: mutual intermingling growth, where both fungi grew into each other without any macroscopic signs of interaction; (2) Type B: mutual inhibition on contact or space between colonies small (< 2 mm); (3) Type C: inhibition of one species on contact, the inhibited species continued to grow at a significantly reduced rate, while the inhibitor species grew at a slightly, reduced rate or unchanged; (4) Type D: mutual inhibition at a distance (>2 mm); (5) Type E: inhibition of one species on contact, the inhibitor species continuing to grow at a reduced rate through the inhibited colony; (6) Type F: inhibition of one species on contact or at a distance, the inhibitor species then continuing to grow at an unchanged rate through or over the inhibited colony” (Wheeler and Hocking, 1993).

### Antifungal non-volatile compounds test

The effects of non-volatile metabolites produced by the selected six endophytic fungi/antagonists were determined using the method described by Hajieghrari et al. (2008). A fungal disc of 5 mm was inoculated into a 250 mL conical flask containing 100 mL of PDB and incubated at 27°C for 15 days, and then filtered through a Whatman filter paper. The filtrate was mixed into molten PDA at a final concentration of 20% (v/v) and poured into Petri dishes. Once solidified, a 5 mm disc of the test pathogen was placed at the center of the PDA plate. The plate was incubated at 27°C. Control plates were prepared without amending PDA with the culture filtrate. After 7 days, radial growth of the test pathogen on the culture filtrate-containing media and control plates were measured, and percentage inhibition in mycelial growth was calculated as explained in the dual culture assay.

### *In Vitro* antibacterial assays

Four bacterial strains (test organisms) were used in this study including two gram-positive bacteria, *Bacillus subtilis* (UPMC 1175) and *Staphylococcus aureus* (ATCC 43300), and two gram-negative bacteria, *Escherichia coli* (ATCC E266) and *Pseudomonas aeruginosa* (ATCC 15442). They were obtained from the Institute of Biosciences (IBS), UPM. Bacterial cultures were grown in nutrient broth (NB) (Friendemann Schmidt, Australia) at 37°C for 24 h before being used in the assays.

### Preliminary screening for antibacterial activity using plate assay

Antibacterial assay was performed according to Onn et al. (2016). Nutrient agar (NA) (Friendemann Schmidt, Australia) plate was prepared and a loopful of the test organism was then streaked onto the plate. A 1 cm disc (agar plug) was cut out from 1-week-old fungal culture, placed on the streaked agar and then incubated at 30°C for 24 h. The assay was replicated 3 times for each bacterium/antagonist combination. Streptomycin was used in place of the fungal agar plug to serve as positive control. The presence of a clear zone using naked eye observation indicates that the fungal isolate possess antibacterial activity, and thus was subjected to the Minimum Inhibitory Concentration (MIC) test. Only identified fungal isolates that showed inhibitory effects toward the bacterial strains in the preliminary screening were selected for subsequent tests.

### Preparation of crude fungal extracts

Two fungal isolates, *F*. *lateritium* and *Xylaria* sp. were selected based on preliminary screenings against fungal pathogen *F. solani* using the dual culture test and the antibacterial plate assay. Actively growing fungal colony on a PDA plate was used as the inoculum. Five fungal discs were cut out from the plate and inoculated into a 500 mL Erlenmeyer flask containing 100 mL of Potato Dextrose Broth (PDB) (Friendemann Schmidt, Australia) at pH 6.5, and allowed to grow in a shaking incubator (120 rpm) at 26°C for 14 days. Then, the liquid culture was filtered using a Whatman filter paper and the filtrate was extracted twice with equal volume of ethyl acetate (R and M Chemicals, Malaysia). The extract was concentrated under reduced pressure at 40°C using a rotary vacuum evaporator and the crude extract was kept at −20°C until being used in the *in vitro* antibacterial and antioxidant assays.

### Determination of minimum inhibitory concentration (MIC)

The MIC test was determined by micro-dilution method under aseptic condition in 96-well plate as described by Daouk et al. (1995). Crude extract of fungal isolates *F*. *lateritium* and *Xylaria* sp. were dissolved in ethyl acetate and working concentrations were prepared in a 2-fold serial dilutions with the highest concentration at 2 mg/mL. Starting with the lowest concentration, 100 uL of the extracts were pipetted into the first row of the plate, and then 50 uL of NB was added into each well, followed by 1 uL of the tested bacteria. For control, 10 mg/mL of Streptomycin (BioBasic Inc, Canada) was used, replacing the fungal crude extract. The plate was incubated overnight at 37°C. After 24 h, 30 μL of 0.02% (w/v) resazurin (Sigma-Aldrich, Germany) was added into each well and the plate was incubated for another 2 h and then subjected to naked eye observation. MIC is defined as the lowest concentration of the extract that inhibits the visible growth of the bacteria, which can be detected by changes in the color of the resazurin, from blue to pink.

### Determination of minimum bactericidal concentration (MBC)

The MBC test was determined by micro-dilution method under aseptic condition in 96-well plate as described by Daouk et al. (1995). From the above mentioned MIC assay, contents from wells with concentrations higher than the MIC value were directly plated on NA (Friendemann Schmidt, Australia) plates. The MBC value was determined when there was no observable colony growth. In addition, the contents from wells with bacterial growth were serially diluted to quantify an end-point killing of the bacteria. Each experiment was performed in triplicates and repeated at three independent times.

### Antioxidant activity

The antioxidant effect of the two fungal isolates *F*. *lateritium* and *Xylaria* sp. were monitored using the 1,1-diphenyl-2-picrylhydrazyl (DPPH) radical scavenging test according to the method described by Liu et al. (2007). Fungal crude extract was dissolved in methanol and working solutions were prepared in five different concentrations in a series of 2-fold dilutions starting at 1,000 μg/mL. 100 μL of the working solution was mixed with 2,900 μL of 120 μM DPPH (Friendemann Schmidt, Australia) in methanol. The mixture was vortexed and incubated at 37°C in the dark for 30 min. Absorbance was recorded at 517 nm using a UV spectrophotometer (GENESYS 10S UV-Vis, USA). Inhibition of free radical by DPPH in percentage (*A*%) was calculated using the following equation: *A*% = [(A_0_-A_i_)/A_0_] × 100; where, A_0_ = absorbance of the control reaction (without test compound), and A_i_ = absorbance of the test compound. Ascorbic acid was used as positive control and all tests were carried out in triplicates. ANOVA was performed using the SAS statistical package (SAS Institute, Cary, NC). For significant effect, the LSD was performed at *P* ≤ 0.05.

## Results

### Endophytic fungi identification

**Figure 1:**
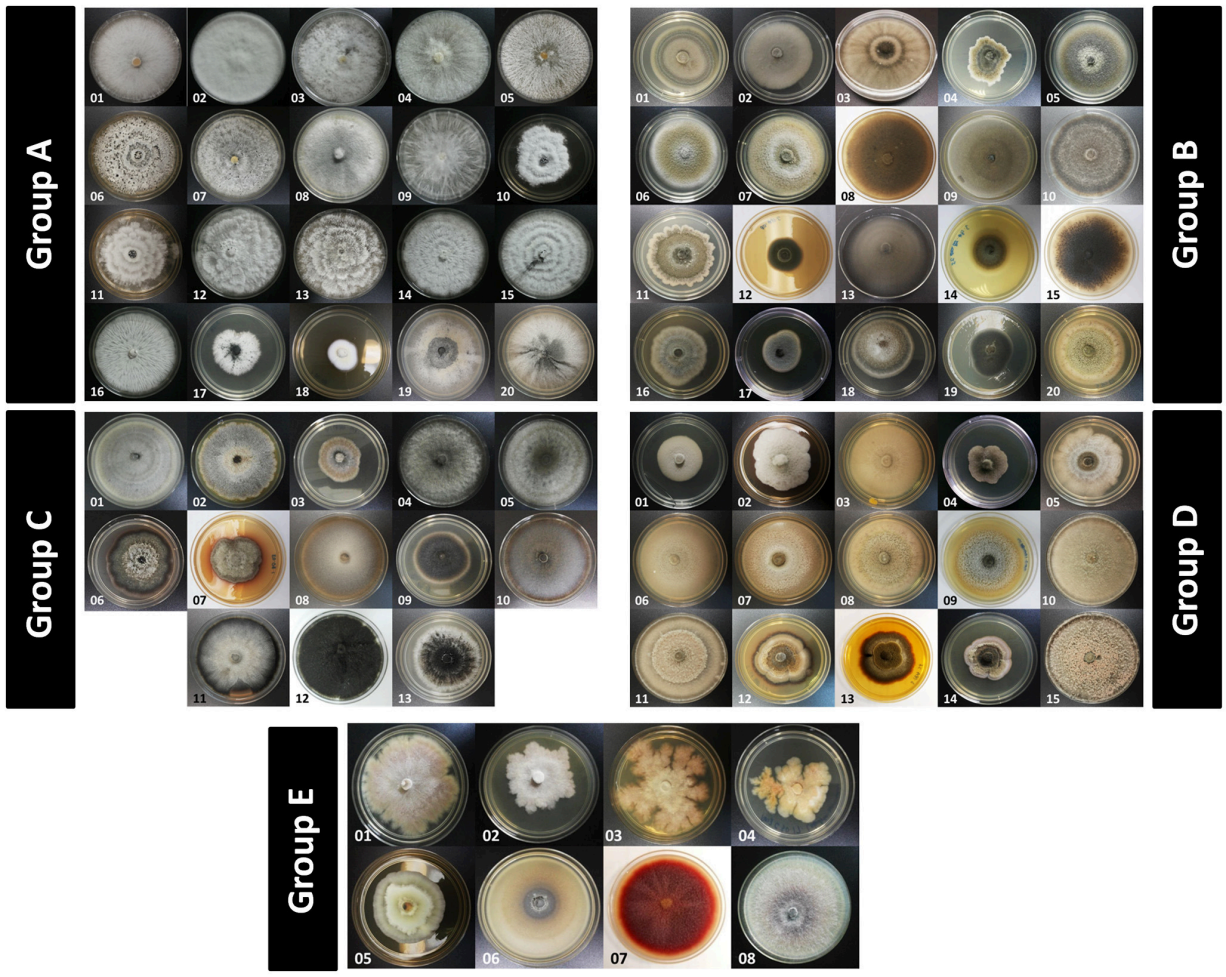
Endophytic fungi isolated from the leaves of *Rhizophora mucronata*. Shown here are some representatives of the fungal isolates, grouped according to their color of colonies and morphological characteristics. All fungal strains were isolated from the inside leaf tissues of the tropical mangrove species and cultivated on conventional media (Medium B) for 7 days at 27°C. Details on the morphological characteristics of the 350 isolates are listed in Supplementary Table 1.

A total of 350 endophytic fungi isolates (Supplementary Table S1) were successfully isolated from 300 leaf segments of *R. mucronata* trees growing in the mangrove forest. However, due to the large number of isolated fungi, they were grouped based on their morphological characteristics (colony form, mycelium color and reverse media color) to allow for a systematic selection of the isolates (Figure 1). The isolates were classified into five groups (A-E) according to their colony color: white, green, gray/black, brown, and others (orange, yellow, purple and violet), respectively. Other characteristics that were taken into account were colony form, colony margin, and pigmentation. Based on this grouping, 101 fungal isolates were chosen for molecular identification, of which only 78 were successfully amplified using primers ITS1 and ITS4. BLAST searches revealed their identities as members of 22 different genera (Table 1), with a majority (96.2%) belonging to the phylum Ascomycota, which includes two classes, Sordariomycetes and Dothideomycetes. On the other hand, members from the phylum Basidiomycota only comprised 3.8% of the total identified specimens. The dominating genera in this study were *Pestalotiopsis* (20.3%), *Alternaria* (10.1%), and *Cladosporium* (6.3%), whereas genera that were represented by a single isolate were *Periconia, Pithomyces*, and *Xylaria*. Other identified genera were *Curvularia, Diaporthe, Epicoccum, Fusarium, Leptosphaeria, Lophiostoma, Nigrospora, Phaeosphaeriopsis, Phoma, Phomopsis, Schizophyllum*, and *Stagonosporopsis*. The ITS sequences obtained through this study were deposited in the NCBI GenBank (Accession no. MH397067-MH397144) for future reference.

**Table 1:** Maximum nucleotide identity match for 78 fungal isolates based on ITS sequences using BLAST analysis.

**No**.	**Isolate ID (GenBank Accession)**	**Match identity (%)**	**E-value**	**Species**	**GenBank Accession**	**Phylum, Class, Family**
1	RM1.18A.01 (MH397067)	100	0.0	*Pestalotiopsis maculans*	KX610327	Ascomycota, Sordariomycetes, Pestalotiopsidaceae
2	RM1.18A.02 (MH397068)	97	0.0	*Diaporthe phaseolorum*	AF001017	Ascomycota, Sordariomycetes, Diaporthaceae
3	RM1.18A.03 (MH397069)	100	0.0	*Neopestalotiopsis egyptiaca*	KP943747	Ascomycota, Sordariomycetes, Pestalotiopsidaceae
4	RM1.18A.04 (MH397070)	100	0.0	*Phaeosphaeriopsis musae*	KR056291	Ascomycota, Dothideomycetes, Phaeosphaeriaceae
5	RM1.18A.05 (MH397071)	100	0.0	*Lophiostoma helminthicola*	JQ026217	Ascomycota, Dothideomycetes, Lophiostomataceae
6	RM1.18A.06 (MH397072)	99	0.0	*Nigrospora oryzae*	KM979813	Ascomycota, Sordariomycetes, Apiosporaceae
7	RM1.18A.07 (MH397073)	99	0.0	*Letendraea helminthicola*	KU529827	Ascomycota, Dothideomycetes, Tubeufiaceae
8	RM1.18A.08 (MH397074)	97	0.0	*Xylaria* sp.	KC507212	Ascomycota, Sordariomycetes, Xylariaceae
9	RM1.18A.09 (MH397075)	99	0.0	*Alternaria alternate*	KY609180	Ascomycota, Dothideomycetes, Pleosporaceae
10	RM1.18A.10 (MH397076)	99	0.0	*Fusarium lateritium*	AY266406	Ascomycota, Sordariomycetes, Nectriaceae
11	RM1.18A.11 (MH397077)	99	0.0	*Pestalotiopsis protearum*	JX556231	Ascomycota, Sordariomycetes, Pestalotiopsidaceae
12	RM1.18A.13 (MH397078)	99	0.0	*Nigrospora oryzae*	HQ608152	Ascomycota, Sordariomycetes, Apiosporaceae
13	RM1.18A.14 (MH397079)	99	0.0	*Nigrospora oryzae*	HQ608152	Ascomycota, Sordariomycetes, Apiosporaceae
14	RM2.18A.01 (MH397080)	99	0.0	*Fusarium proliferatum*	KR350649	Ascomycota, Sordariomycetes, Nectriaceae
15	RM2.18A.02 (MH397081)	100	0.0	*Neopestalotiopsis egyptiaca*	KP943747	Ascomycota, Sordariomycetes, Pestalotiopsidaceae
16	RM2.18A.03 (MH397082)	100	0.0	*Neopestalotiopsis egyptiaca*	KP943747	Ascomycota, Sordariomycetes, Pestalotiopsidaceae
17	RM2.18A.06 (MH397083)	98	0.0	*Fusarium verticillioides*	KR183784	Ascomycota, Sordariomycetes, Nectriaceae
18	RM2.18A.07 (MH397084)	100	0.0	*Fusarium proliferatum*	KR350649	Ascomycota, Sordariomycetes, Nectriaceae
19	RM3.18A.01 (MH397085)	99	0.0	*Pestalotiopsis protearum*	JX556231	Ascomycota, Sordariomycetes, Pestalotiopsidaceae
20	RM3.18A.02 (MH397086)	99	0.0	*Pestalotiopsis protearum*	JX556231	Ascomycota, Sordariomycetes, Pestalotiopsidaceae
21	RM3.18A.03 (MH397087)	99	0.0	*Neopestalotiopsis egyptiaca*	KP943747	Ascomycota, Sordariomycetes, Pestalotiopsidaceae
22	RM3.18A.04 (MH397088)	99	0.0	*Neopestalotiopsis clavispora*	KY319134	Ascomycota, Sordariomycetes, Pestalotiopsidaceae
23	RM3.18A.05 (MH397089)	99	0.0	*Schizophyllum commune*	KP202299	Basidiomycota, Agaricomycetes, Schizophyllaceae
24	RM3.18A.06 (MH397090)	99	0.0	*Schizophyllum commune*	AB369909	Basidiomycota, Agaricomycetes, Schizophyllaceae
25	RM3.18A.07 (MH397091)	99	0.0	*Phomopsis* sp.	KX020566	Ascomycota, Sordariomycetes, Diaporthaceae
26	RM3.18A.08 (MH397092)	99	0.0	*Cladosporium perangustum*	KP701968	Ascomycota, Dothideomycetes, Cladosporiaceae
27	RM3.18A.09 (MH397093)	100	0.0	*Cladosporium tenuissimum*	KP701937	Ascomycota, Dothideomycetes, Cladosporiaceae
28	RM3.18A.10 (MH397094)	93	0.0	*Periconia pseudobyssoides*	KC954161	Ascomycota, Dothideomycetes
29	RM3.18A.11 (MH397095)	100	0.0	*Arthrinium* sp.	HQ022507	Ascomycota, Sordariomycetes, Apiosporaceae
30	RM3.18A.13 (MH397096)	100	0.0	*Phaeosphaeriopsis musae*	KR056291	Ascomycota, Dothideomycetes, Phaeosphaeriaceae
31	RM3.18A.14 (MH397097)	99	0.0	*Curvularia lunata*	DQ836800	Ascomycota, Dothideomycetes, Pleosporaceae
32	RM3.18A.15 (MH397098)	99	0.0	*Epicoccum nigrum*	KM507768	Ascomycota, Dothideomycetes, Pleosporaceae
33	RM3.18A.16 (MH397099)	100	0.0	*Phoma* sp.	KT150683	Ascomycota, Dothideomycetes, Didymellaceae
34	RM3.18A.17 (MH397100)	99	0.0	*Stagonosporopsis cucurbitacearum*	KU059901	Ascomycota, Dothideomycetes, Didymellaceae
35	RM3.18A.18 (MH397101)	100	0.0	*Curvularia brachyspora*	HG778983	Ascomycota, Dothideomycetes, Pleosporaceae
36	RM3.18A.20 (MH397102)	99	0.0	*Stagonosporopsis cucurbitacearum*	KU059901	Ascomycota, Dothideomycetes, Didymellaceae
37	RM3.18A.23 (MH397103)	99	0.0	*Paraphaeosphaeria* sp.	KX611058	Ascomycota, Dothideomycetes, Didymosphaeriaceae
38	RM3.18A.24 (MH397104)	99	0.0	*Pithomyces maydicus*	HG933803	Ascomycota, Dothideomycetes, Pleosporaceae
39	RM3.18A.25 (MH397105)	100	0.0	*Arthrinium* sp.	HQ022507	Ascomycota, Sordariomycetes, Apiosporaceae
40	RM3.18A.26 (MH397106)	100	0.0	*Phoma* sp.	KT150683	Ascomycota, Dothideomycetes, Didymellaceae
41	RM1.30.01 (MH397107)	99	0.0	*Stagonosporopsis cucurbitacearum*	KU059901	Ascomycota, Dothideomycetes, Didymellaceae
42	RM1.30.02 (MH397108)	99	0.0	*Epicoccum nigrum*	KR094452	Ascomycota, Dothideomycetes, Pleosporaceae
43	RM1.30.04 (MH397109)	99	0.0	*Alternaria eichhorniae*	KU593527	Ascomycota, Dothideomycetes, Pleosporaceae
44	RM1.30.05 (MH397110)	100	0.0	*Neopestalotiopsis egyptiaca*	KP943747	Ascomycota, Sordariomycetes, Pestalotiopsidaceae
45	RM1.30.07 (MH397111)	100	0.0	*Alternaria eichhorniae*	KU593527	Ascomycota, Dothideomycetes, Pleosporaceae
46	RM1.30.08 (MH397112)	99	0.0	*Epicoccum nigrum*	HQ728258	Ascomycota, Dothideomycetes, Pleosporaceae
47	RM1.30.09 (MH397113)	99	0.0	*Epicoccum nigrum*	HQ728258	Ascomycota, Dothideomycetes, Pleosporaceae
48	RM1.30.11 (MH397114)	99	0.0	*Neopestalotiopsis protearum*	KT936426	Ascomycota, Sordariomycetes, Pestalotiopsidaceae
49	RM1.30.12 (MH397115)	100	0.0	*Neopestalotiopsis protearum*	KX631739	Ascomycota, Sordariomycetes, Pestalotiopsidaceae
50	RM1.30.13 (MH397116)	99	0.0	*Neopestalotiopsis egyptiaca*	KP943747	Ascomycota, Sordariomycetes, Pestalotiopsidaceae
51	RM1.30.15 (MH397117)	100	0.0	*Alternaria arborescens*	KP942903	Ascomycota, Dothideomycetes, Pleosporaceae
52	RM2.30.02 (MH397118)	100	0.0	*Lophiostoma helminthicola*	JN116664	Ascomycota, Dothideomycetes, Lophiostomataceae
53	RM2.30.03 (MH397119)	99	0.0	*Neopestalotiopsis foedans*	KU593530	Ascomycota, Sordariomycetes, Pestalotiopsidaceae
54	RM2.30.05 (MH397120)	100	0.0	*Phoma leveillei*	KT963795	Ascomycota, Dothideomycetes, Didymellaceae
55	RM2.30.07 (MH397121)	100	0.0	*Curvularia affinis*	HG778982	Ascomycota, Dothideomycetes, Pleosporaceae
56	RM2.30.11 (MH397122)	100	0.0	*Schizophyllum commune*	KP202299	Basidiomycota, Agaricomycetes, Schizophyllaceae
57	RM2.30.12 (MH397123)	100	0.0	*Cladosporium cladosporioides*	JX230994	Ascomycota, Dothideomycetes, Cladosporiaceae
58	RM2.30.15 (MH397124)	100	0.0	*Phoma herbarum*	MF120206	Ascomycota, Dothideomycetes, Didymellaceae
59	RM2.30.17 (MH397125)	99	0.0	*Pestalotiopsis* sp.	KR085971	Ascomycota, Sordariomycetes, Pestalotiopsidaceae
60	RM3.30.01 (MH397126)	99	0.0	*Diaporthe yunnanensis*	KY491542	Ascomycota, Sordariomycetes, Diaporthaceae
61	RM3.30.02 (MH397127)	99	0.0	*Leptosphaeria* sp.	KM979814	Ascomycota, Dothideomycetes, Leptosphaeriaceae
62	RM3.30.04 (MH397128)	99	0.0	*Alternaria macrospora*	AY154689	Ascomycota, Dothideomycetes, Pleosporaceae
63	RM3.30.05 (MH397129)	99	0.0	*Phaeosphaeriopsis* sp.	KR012892	Ascomycota, Dothideomycetes, Phaeosphaeriaceae
64	RM3.30.06 (MH397130)	100	0.0	*Arthrinium* sp.	HQ022507	Ascomycota, Sordariomycetes, Apiosporaceae
65	RM3.30.07 (MH397131)	100	0.0	*Cladosporium tenuissimum*	KP701937	Ascomycota, Dothideomycetes, Cladosporiaceae
66	RM3.30.08 (MH397132)	100	0.0	*Cladosporium cladosporioides*	KM980007	Ascomycota, Dothideomycetes, Cladosporiaceae
67	RM3.30.09 (MH397133)	99	0.0	*Leptosphaeria* sp.	KM979814	Ascomycota, Dothideomycetes, Leptosphaeriaceae
68	RM3.30.10 (MH397134)	99	0.0	*Periconia byssoides*	MF435088	Ascomycota, Dothideomycetes
69	RM3.30.12 (MH397135)	99	0.0	*Paraphaeosphaeria* sp.	KX611058	Ascomycota, Dothideomycetes, Didymosphaeriaceae
70	RM3.30.13 (MH397136)	100	0.0	*Lophiostoma helminthicola*	JN116664	Ascomycota, Dothideomycetes, Lophiostomataceae
71	RM3.30.14 (MH397137)	100	0.0	*Arthrinium* sp.	HQ022507	Ascomycota, Sordariomycetes, Apiosporaceae
72	RM3.30.15 (MH397138)	99	0.0	*Leptosphaeria* sp.	KM979814	Ascomycota, Dothideomycetes, Leptosphaeriaceae
73	RM3.30.16 (MH397139)	100	0.0	*Alternaria arborescens*	KP942903	Ascomycota, Dothideomycetes, Pleosporaceae
74	RM3.30.17 (MH397140)	100	0.0	*Letendraea helminthicola*	KP263123	Ascomycota, Dothideomycetes, Tubeufiaceae
75	RM3.30.18 (MH397141)	100	0.0	*Alternaria arborescens*	KP942903	Ascomycota, Dothideomycetes, Pleosporaceae
76	RM3.30.19 (MH397142)	99	0.0	*Curvularia eragrostidis*	HG778986	Ascomycota; Dothideomycetes, Pleosporaceae
77	RM3.30.20 (MH397143)	99	0.0	*Leptosphaeria* sp.	KM979814	Ascomycota, Dothideomycetes, Leptosphaeriaceae
78	RM3.30.22 (MH397144)	100	0.0	*Curvularia lunata*	HQ607991	Ascomycota, Dothideomycetes, Pleosporaceae

**Figure 2:**
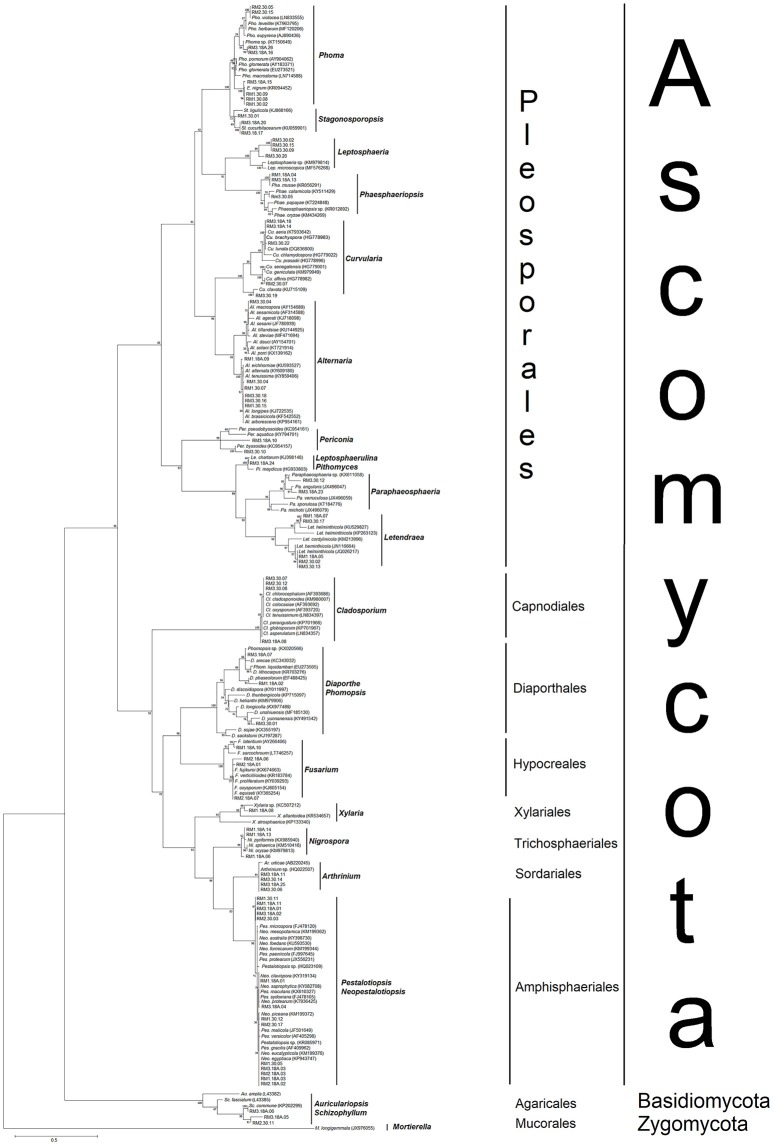
Maximum likelihood (ML) phylogenetic tree based on rDNA ITS sequences of endophytic fungal isolates and fungal ITS sequences from the GenBank. ML tree was constructed using the substitution model general time reversible (GTR) model and gamma distributed (+G) with invariant sites (+I) (= GTR+G+I). All positions containing gaps and missing data were included for analysis. Clade supports were calculated based on 1000 bootstrap re-samplings. Accession numbers of the 78 sequences belonging to the endophytic fungi isolated in this study are listed in Table 1, while the reference sequences (117) from the GenBank are listed in Supplementary Table 2.

A total of 117 sequences of close relatives (Supplementary Table S2) were downloaded from the NCBI GenBank and combined with sequences of the 78 endophytes for phylogenetic tree construction (Figure 2). Nine different orders were observed, of which seven representing Ascomycota, and one order each representing Basidiomycota and the outgroup Zygomycota. Most of the fungal isolates clustered under the order Pleosporales, belonging to at least 12 different genera. Among the 78 isolates obtained from this study, eight (RM3.18A.15, RM3.18A.16, RM3.18A.26, RM1.30.02, RM1.30.08, RM1.30.09, RM2.30.05, and RM2.30.15) were grouped under the *Phoma*-*Epicoccum* clade with 100% bootstrap support (Figure 2). Similarly, other identified isolates clustered into clades of known sequences and received high bootstrap support (91–100%). For example, seven isolates (RM3.30.04, RM1.18A.09, RM1.30.04, RM1.30.07, RM3.30.18, RM3.30.16, and RM1.30.15) identified from their sequences as closely related to *Alternaria* sp., clustered into their predicted clade (99%). For the order Capnodiales, four isolates (RM3.30.07, RM2.30.12, RM3.30.08, and RM3.18A.08) clustered under *Cladosporium* (100%) and for Hypocreales, four isolates (RM1.18A.10, RM2.18A.06, RM2.18A.01, and RM2.18A.07) clustered with *Fusarium* (100%). For the order Xylariales, RM1.18A.08 was clustered under the *Xylaria* clade with 92% bootstrap support.

In Trichospaeriales, three isolates (RM1.18A.06, RM1.18A.13, and RM1.18A.14) clustered under the *Nigrospora* clade (99%). Four isolates (RM3.18A.11, RM3.18A.25, RM3.30.04, and RM3.30.06) clustered under *Athrinium* of Sordariales. The order with the most isolates obtained from this study was Amphisphaeriales. The cluster was constructed within a polytomy consisting *Pestalotiopsis* and *Neopestalotiopsis* (98%), recorded with 14 isolates (RM1.18A.01, RM1.18A.11, RM1.18A.03, RM2.18A.02, RM2.18A.03, RM3.18A.01, RM3.18A.02, RM3.18A.03, RM3.18A.04, RM1.30.05, RM1.30.11, RM1.30.12, RM2.30.03, and RM2.30.17). As for the phylum Basidiomycota, three isolates (RM2.30.11, RM3.18A.05 and RM3.18A.06) were clustered under the Auriculariopsis-Schizophyllum clade, showing sister relationship with *S. commune*.

### Fungal interaction during dual culture assay

**Figure 3:**
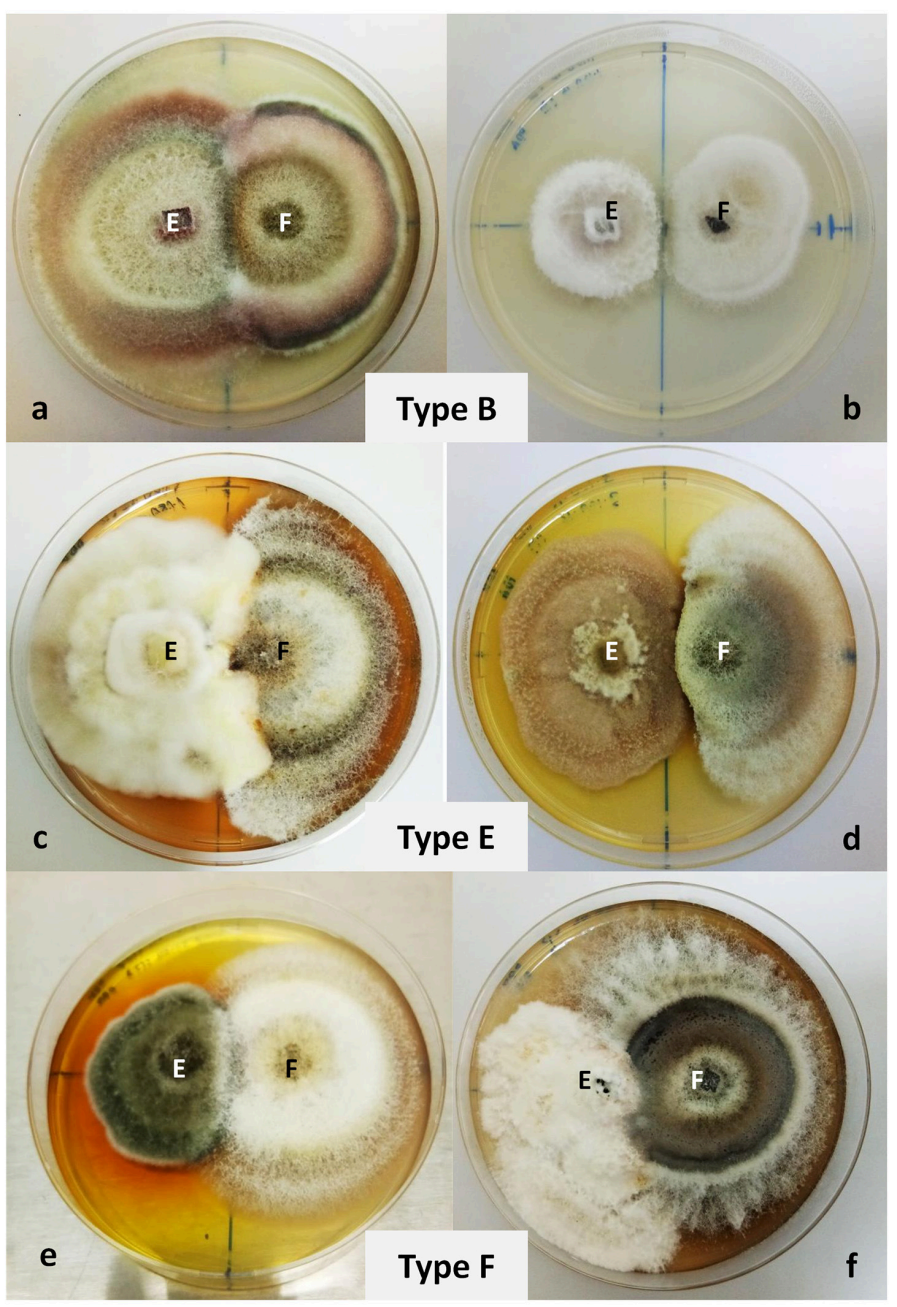
Dual culture plate assay between six endophytic fungal isolates against the pathogen *Fusarium solani*. *Fusarium solani* was grown in PDA plates together with **(a)** *Fusarium lateritium*, **(b)** *Nigrospora oryzae*, **(c)** *Xylaria* sp., **(d)** *Phoma* sp., **(e)** *Alternaria macrospora*, and **(f)** *Pestalotiopsis* sp. The plates were cultivated for 7 days at 27°C. Radial growths were measured and types of interaction were observed. Type B interaction was observed for *F*. *lateritium* and *N*. *oryzae*, type E for *Xylaria* sp. and *Phoma* sp., and type F for *A*. *macrospora* and *Pestalotiopsis* sp.

Six fungal isolates were selected for antagonism assay on the basis of their distinct morphology such as pigment production, indicating their potential in possessing significant bioactive compounds, and based on previous literature reporting on fungal genera with strong bioactivities against other microorganisms (Oliveira et al., 2012; Zhao et al., 2012a). The isolates were RM1.18A.08, RM1.18A.10, RM1.18A.13, RM3.18A.16, RM2.30.17, RM3.30.04, and identified through NCBI Blast match identity as *Xylaria* sp., *Fusarium lateritium, Nigrospora oryzae, Phoma* sp., *Pestalotiopsis* sp., and *Alternaria macrospora*, respectively. From the dual culture plates, we observed 3 different fungal interactions (Wheeler and Hocking, 1993) between the isolated fungal antagonists and the pathogen *F. solani*. Both *F. lateritium* and *N*. *oryzae* exhibited type B interaction when cultured with *F. solani*. The fungal endophytes and pathogen displayed a mutual inhibition on contact (Figures 3a,b). However, in the dual culture plate of *N*. *oryzae* and *F*. *solani*, a small clear zone (>2 mm) was recorded. In *Xylaria* sp./*F. solani* interaction, the mycelium of the antagonist had breached into the pathogen's colony. Yellowish pigmentation was first observed at the border of the two colonies, which later turned into rust after several days (Figure 3c). In *Phoma* sp./*F. solani* interaction, dark purple pigmentation was observed on the test pathogen. This combination exhibited type E interaction—growth of the test endophyte was unaffected while that of the pathogen was reduced (Figure 3d). For *A*. *macrospora* and *Pestalotiopsis* sp., they both showed type F interaction when co-cultivated with *F. solani* (Figures 3e,f). Growth of the endophytes were inhibited by the pathogen, which eventually overgrown the inhibited colony. The *A*. *macrospora* colony had been producing yellow pigments (data not shown) beginning on the third day after inoculation, which then turned into dark brown on the seventh day (Figure 3e).

### Antagonistic activity of the fungal isolates against *Fusarium solani*

Dual culture assay revealed the inhibition percentage (*I*%) of each endophytic fungus against the pathogen *F. solani* (Table 2). *Phoma* sp. recorded the highest *I*% at 69.64%. *Xylaria* sp. the second highest *I*% (65.11%), followed by *N. oryzae* (64.03%), and *F. lateritium* (61.89%). Mycelium development of *F. solani* in dual culture plates was significantly inhibited (*F* test = 56.31, *p* < 0.0001). There was significant differences between means of inhibition by *Phoma* sp. when compared to the other five species, while no differences were observed between *Xylaria* sp. and *N*. *oryzae*, and *N*. *oryzae* with *F*. *lateritium*. Their antagonistic ability to produce non-volatile compounds to inhibit the test pathogen was further confirmed. Radial growth of *F*. *solani* mycelia in plates containing the endophytic fungal filtrates showed significant *I*% for *Xylaria* sp. (25.07%), *Phoma* sp. (23.36%), and *F*. *lateritium* (21.19%) (*F* = 63.71, *p* < 0.0001) (Table 2).

**Table 2:** *In vitro* antagonism of six selected endophytic fungal isolates against the pathogenic fungus, *Fusarium solani*, using two types of assays.

**Isolate name**	**Percent inhibition growth over control (*****I*****%)** ± **SD**	
	**Dual culture assay^A^**	**Non-volatile compound assay^B^**
*Phoma* sp.	69.64^a^ ± 1.76	23.26^a, c^ ± 3.31
*Xylaria* sp.	65.11^b^ ± 0.91	25.07^a, b^ ± 1.61
*Nigrospora oryzae*	64.03^b, c^ ± 0.40	2.07^d^ ± 3.58
*Fusarium lateritium*	61.89^c^ ± 2.05	21.19^b, c^ ± 0.45
*Alternaria macrospora*	59.05^d^ ± 1.61	1.29^d^ ± 1.19
*Pestalotiopsis* sp.	49.40^e^ ± 1.61	11.63^e^ ± 2.05

*Data are means of percentage of growth inhibition ± standard deviation (SD). Means followed by the same letter in each group are not significantly different at α = 0.05 according to Tukey test (LSD = 2.83 and 4.11, for A and B assays, respectively)*.

### Antibacterial properties

From the preliminary qualitative antibacterial screening of the six fungal isolates, *F*. *lateritium* showed inhibition to all four bacterial pathogens tested, while *Xylaria* sp. to three (Table 3). However, the other four endophytic isolates including the high performer from the *in vitro* antagonistic assays, *Phoma* sp., gave no inhibition effect to any of the tested bacteria. Ethyl acetate-extracts of the fungi were used to determine the minimum concentration required to inhibit bacterial growths. For these two fungi, crude extract at 0.50 mg/mL was sufficient to inhibit *E. coli* and *P. aeruginosa*; at 1.00 mg/mL was needed to inhibit *S. aureus*, and the highest concentration at 2.00 mg/mL was required for *B. subtilis* (Table 4).

**Table 3:** Preliminary screening for antibacterial activities in six selected endophytic fungal isolates against *Bacillus subtilis, Escherichia coli, Pseudomonas aeruginosa*, and *Staphylococcus aureus* using plate assay.

**Isolates**	***Staphylococcus aureus ATCC 43300***			***Escherichia coli ATCC 25922***			***Pseudomonas aeruginosa ATCC 15542***			***Bacillussubtilis B29***		
	**i**	**ii**	**iii**	**i**	**ii**	**iii**	**i**	**ii**	**iii**	**i**	**ii**	**iii**
*Alternaria macrospora*	–	–	–	–	–	–	–	–	–	–	–	–
*Fusarium lateritium*	+	–	+	+	+	–	+	–	–	+	+	–
*Nigrospora oryzae*	–	–	–	–	–	–	–	–	–	–	–	–
*Pestalotiopsis* sp.	–	–	–	–	–	–	–	–	–	–	–	–
*Phoma* sp.	–	–	–	–	–	–	–	–	–	–	–	–
*Xylaria* sp.	–	–	–	+	+	–	+	+	+	+	+	–
Streptomycin	35	35	35	30	30	30	35	35	35	30	30	30

“*+” indicates the fungal isolate inhibited bacterial growth, and “–” indicates no inhibition. Streptomycin was used as positive control*.

**Table 4:** Determination of the Minimum Inhibitory Concentration (MIC) and Minimum Bactericidal Concentration (MBC) of ethyl-acetate extracts of two endophytic fungal isolates against several pathogenic bacteria using the microdilution method.

**Isolates**	**MIC / MBC concentration (mg/mL)**			
	***Bacillus subtilis***	***Escherichia coli***	***Pseudomonas aeruginosa***	***Staphylococcus aureus***
*Fusarium lateritium*	2.00/2.00	0.50/0.50	0.50/0.50	1.00/1.00
*Xylaria* sp.	2.00/2.00	0.50/0.50	0.50/0.50	1.00/1.00

### Antioxidant activity

For antioxidant activity, methanol-extracts of *F. lateritium* and *Xylaria* sp. were evaluated by DPPH radical scavenging assay. The extracts had significant radical scavenging capacity with significant *F* test (*F* = 57.05, *p* < 0.0001). The methanol-extract from *Xylaria* sp. notably reduced the absorbance of DPPH free radical (Table 5). It recorded significantly high scavenging activity (*A*%) of over 90% at the two highest extract concentrations (500 and 1,000 μg/mL) (Table 5). The *A*% was reduced to almost half at 250 μg/mL, and further lowered with decreasing concentrations. In contrast, the methanol-extract of *F. lateritium* had free radicals inhibition activity of only 29.95% at the highest concentration (1,000 μg/mL) and 6.61% at the lowest concentration (62.5 μg/mL), which were significantly lower when compared to *Xylaria* sp. and the positive control, ascorbic acid.

**Table 5:** Antioxidant activity of *Fusarium lateritium* and *Xylaria* sp. as determined from their free radical-scavenging capacity measured by DPPH assay.

**Extract concentration (μg/mL)**	**Inhibition of free radical by DPPH in percentage (*****A*****%)** ± **SD (%)**		
	***Fusarium lateritium***	***Xylaria* sp**.	**Ascorbic acid**
1,000	29.95^c^±0.373	95.82^a^±0.079	61.16^b^±0.518
500	14.03^c^±0.879	91.89^a^±0.925	56.82^b^±4.57
250	8.03^c^±2.567	51.32^b^±0.356	53.03^a^±7.906
125	6.74^c^±1.59	30.32^b^±1.060	47.45^a^±9.733
62.5	6.61^c^±0.273	18.76^b^±1.292	39.61^a^±2.739

*The fungal extract was prepared in a series of 2-fold dilutions. Ascorbic acid served as positive control. Data are means of inhibition of free radicals by DPPH. Means followed by the same letter across each concentration are not significantly different at α = 0.05 according to Tukey test (LSD = 9.07)*.

## Discussion

### Endophytic fungal diversity

From this study, ITS sequences identified 75 fungal isolates representing the phylum Ascomycota, and three from Basidiomycota. The phylum Ascomycota is reportedly the most common representative of endophytic fungi community when isolated using standard isolation protocol (Crozier et al., 2006; He et al., 2012; Koukol et al., 2012), and the same finding was reflected in our study. Fungi from the phylum Basidiomycota have been reported to be culture-method dependent (Crozier et al., 2006), which explains the small number of Basidiomycota isolates in this study. Comparative studies have also indicated that only a small portion of the microorganisms in nature was amenable to cultivation using conventional microbiological techniques (Amann et al., 1995). There are many unfavorable factors that could affect the ability to grow microorganisms in laboratory conditions. Among them are lack of knowledge about their nutritional requirements and demanding nature of the microorganisms specifically when they originate from environmental samples.

Based on the total fungal isolates identified using the DNA method, the genus *Pestalotiopsis* was the most frequent, consisting 20.25% of total fungal isolates obtained from this study. *Pestalotiopsis* as a dominant fungal endophyte has been reported in other plants such as *Carapa guianensis, Catostemma fragrans, Chlorocardium rodiei*, and *Eperua falcata* (Cannon and Simmons, 2002). *Pestalotiopsis* is a beneficial member of the foliar endophytic community because it is capable to switch its nutritional-mode, allowing itself to stay as an endophyte or become a saprobe when necessary (Maharachchikumbura et al., 2012; Douanla-Meli et al., 2013). Besides *Pestalotiopsis*, other fungal genera such as *Alternaria, Cladosporium, Curvularia*, and *Fusarium* were also reportedly predominant mangrove endophytic fungi (Udayanga et al., 2011) and are widespread in different mangrove species (Cheng et al., 2009). Meanwhile, the single isolate genus *Xylaria* is a common endophytic fungal genus found in trees (Suryanarayanan et al., 2002). Although it is widely available in most tropical plants investigated in past studies, we recorded a low isolation frequency for this genus (Chen et al., 2013; Douanla-Meli et al., 2013). Our finding also revealed lesser-known fungal genera, namely *Periconia, Phaeosphaeriopsis, Pithomyces*, and *Stagonosporopsis* as community members in the leaf of *R. mucronata*.

### Potential bioactivities

#### Antagonism properties

Antagonism has been defined as “to include any activity of one organism which in some way adversely affects another growing in association with it” (Khara and Hadwan, 1990). There are several types of antagonism that fungal antagonists could exhibit, such as competition, mycoparasitism, and the production of extracellular metabolites (Siameto et al., 2010). These metabolites, namely antibiotics and lytic enzymes, have been widely applied in various fields such as crop-pathogen controls. Endophytic microorganisms isolated from plants were also reported to be capable of producing a variety of novel bioactive metabolites that are source for novel secondary metabolites (Firáková et al., 2007; Ramasamy et al., 2010). Therefore, attention can be given to the bioactive compounds produced by plants, microbes, and marine organisms for the discovery of compounds useful for the development of new drugs.

*Fusarium solani* is a common tree pathogen and has been isolated from diseased mangrove plants (Umechuruba, 2005). We showed that endophytes *F*. *lateritium, N*. *oryzae, Phoma* sp., and *Xylaria* sp., successfully inhibited the pathogenic *F*. *solani* in the dual culture assay (Figure 3d). The ability to out-grow the pathogenic fungus suggested that *Phoma* sp. inhibited growth of *F*. *solani* by competing for space and nutrients. Spreading of *F*. *solani* was further restricted when mycelium of the two fungi came into contact. Biological agents with antifungal properties are known to secrete certain enzymes, which are responsible for the breaking down of the pathogen's cell wall, thus restricting its growth (Sharon et al., 2001). Antagonist mechanism displayed by *Xylaria* sp. was more aggressive when compared to *Phoma* sp. as it advances and further colonized over the surface of the pathogen (Figure 3c). This phenomenon could be attributed to the production of lytic enzymes by *Xylaria* sp., which degrade the pathogen's cell wall and allows *Xylaria* sp. to colonize the surface of the pathogen's colony, similar to what has been reported in *Streptomyces griseus* (Anitha and Rabeeth, 2010). The antagonistic activity displayed by *Phoma* sp. and *Xylaria* sp. could be explained by their secreting secondary metabolites into the agar, which are detrimental to the pathogen's growth. Arguably, such inhibition could also be due to nutrient depletion in the media (Robinson et al., 2014). Variations in fungal interactions might also be influenced by antibiotics produced from the isolates, which could be fungicidal to a single fungus, but fungistatic to other fungi (Dharmaputra, 2003). Meanwhile, a small inhibition zone >2 mm, was recorded in the dual culture plate of *N*. *oryzae/F*. *solani* (Figure 3b). It could be due to the production of hydrolytic enzymes by certain fungal isolates, or the production of antibiotics by the antagonists (Kamala and Indira, 2011). The various types of interactions we observed are dependent on the specific fungal species combination, because of differences in compounds of the volatile mixture, substrates, or abilities to detoxify the volatile metabolites such as enzymatic activity (Kai et al., 2007). Compound isolation from the endophytic *Nigrospora* sp., has revealed griseofulvin responsible for its strong antifungal activity against a plant pathogenic fungus, *Botrytis cinerea* (Zhao et al., 2012a). Griseofulvin has been widely used to treat pathogenic fungi causal diseases, acting as antibiotics (Zhao et al., 2012a). Many fungi including *Xylaria* sp. have been reported to be typical griseofulvin-producing fungi (Park et al., 2005).

Over time during the dual culture assay, we observed several fungi had produced pigmentation, including *Xylaria* sp., *A*. *macrospora*, and the pathogen *F*. *solani* (Figure 3). Fungi are known to produce a range of bioactive compounds that hold significant roles in the diversification and adaptability of these micro-organisms in various ecological niches (Fox and Howlett, 2008; da Costa Souza et al., 2016). Among the compounds induced by fungi, pigments have been accentuated as the contributor to biological activities exhibited by the fungi (dos Reis Celestino et al., 2014). There are a variety of pigments produced by fungi including carotenoids, melanins, flavins, phenazines, and quinos (Mapari et al., 2010; Dufossé et al., 2014). These pigments are attributed to crucial activities such as antibacterial, antifungal, and herbicidal (Geweely, 2011; Premalatha et al., 2012; Teixeira et al., 2012). This was shown in the present study, where yellow pigmentation was produced by *Xylaria* sp. in the dual culture plate against the pathogen *F*. *solani* (Figure 3c). The same condition was observed for isolate *A*. *macrospora*, where it started producing yellow pigmentation since day three of the dual culture assay. The yellowish pigmentation turned darker, eventually into brown, after 7 days. While, in the case of *Phoma* sp./*F*. *solani*, the pathogen started producing dark purple pigmentation when their mycelia got in contact. Many fungi produce extracellular pigments in response to adverse conditions, such as low moisture, pH, and UV light, or as a way to protect their resources from other fungi (Score et al., 1997; Tudor et al., 2013). Under natural conditions, when the supplies of vital nutrients become depleted, parts of the fungal mycelium will switch their biochemical activity to the pathways of secondary metabolism (Isaac, 1994). Rather than producing new fungal building material, these fungi start to produce metabolites manifested as pigmentation. The production of the pigments might have resulted from the defense mechanism of the fungi to protect their mycelia from being hydrolyzed by the enzymes produced by other microbes (Isaac, 1994).

Production of antimicrobial compounds is one of the main mechanisms employed by antagonists for pathogen control. The antimicrobial compounds may be produced in the form of volatiles and non-volatiles (Cazar et al., 2005). One of the simplest and most effective method to identify the potential antagonists against pathogen is by the non-volatile compound test. In this study, we examined six fungal isolates for their production of non-volatile compounds that could inhibit the growth of *F*. *solani in vitro*. Based on the results, it is evident that the non-volatile compounds suppressed the pathogen's growth, causing mycelial growth reduction in *F*. *solani* when compared to the control plates. The culture filtrates from these isolates displayed inhibitory effects on the radial growth of the pathogen, and the mycelia accumulation observed in this study indicated the presence of non-volatile antibiotics in the filtrate. Antibiotic inhibitions have been reportedly useful in reducing plant diseases in previous studies (Dubey and Suresh, 2006).

#### Antibacterial activities

Since the last three decades, pathogenic microbes have become more resistant to modern antibiotics (Cui et al., 2015). It was reported that the antibacterial activities exhibited by leaf extracts of several mangrove species such as *Acanthus ilicifolius, Excoecaria agallocha, R. mucronata*, and *Sonneratia caseolaris*, showed significant inhibition on the growth of multidrug resistance pathogenic bacteria *Staphylococcus aureus* (Prihanto et al., 2011). We observed similar findings through this study. The enormous diversity of endophytic fungi of the mangrove species was believed to be the major factor. Previously, it was reported that *Xylaria* sp. contained 7-amino-4-methylcoumarin compound, which was proven to exhibit strong inhibitory activities against 13 microorganisms, including *E*. *coli* and *S*. *aureus* (Liu et al., 2008). Other genera which also showed effectiveness in inhibition of microbial growth were *Aspergillus, Botryosphaeria, Eutypella, Fusarium, Guignardia, Penicillium and Phomopsis* (Phongpaichit et al., 2006; Bernardi-Wenzel et al., 2010; Rhoden et al., 2012). Therefore, it could be suggested that the antibacterial activities exhibited in mangrove leaf extracts were probably contributed by the endophytic fungi living in them. This claim was supported by the findings of a group of Chinese researchers who isolated 130 endophytic fungi from several Chinese medicinal plants, and further tested the antitumor and antifungal activities from their extracts. As a result, 9.2% of the extracts were positive in antitumour activity, with 30% of the endophytes extracted from the plants exhibited antifungal activity, thus indicating presence in fungal compounds that could be associated with the host plants (Li et al., 2005). In addition, it was also reported that endophytic fungi could produce bioactive compounds associated with their host plants (Kusari et al., 2013).

In the recent study, *F*. *lateritium* was shown to successfully inhibit all four pathogenic bacteria, with low MIC values. Endophytic *Fusarium* species of medicinal plants are well-known sources of bioactive compounds including against microbial activity (Liang et al., 2012). Several compounds that were proven to exhibit antimicrobial activities include pentaketide (2-methylbutyraldehyde-substituted-α-pyrone) isolated from *Fusarium* sp. of *Selaginella pallescens*, which showed strong activity against *Candida albicans* (Brady and Clardy, 2000); beauvericin from *F. oxysporum*, isolated from the bark of *Cinnamomum kanehirae*, which significantly suppressed the growth of methicillin-resistant bacteria, *B*. *subtillis* and *S*. *aureus* (Wang et al., 2011); and subglutinol A and B, from *Fusarium* sp. of a medicinal plant *Tripterygium wilfordii* (Lee et al., 1995). Another compound equisetin, extracted from *Fusarium* sp., was not only responsible for the antimicrobial activity, but also aided the plant to successfully withstand stressful environmental conditions (Ratnaweera et al., 2015).

#### Antioxidant activity

The DPPH test showed that the methanol extract of *F*. *lateritium* had a lower antioxidant activity (6.6–29.9) in contrast to that of *Xylaria* sp. (18.7–95.8%) (Table 5). At the concentration of 1,000 μg/mL, *Xylaria* sp. displayed higher scavenging activity even above that of the positive control, ascorbic acid (61.6%). The activity was observed to be extract concentration-dependent, with scavenging activity directly proportional to the concentration. This is noticeable by the color change in the solution. The big difference between these two isolates could be due to the high compound content in *Xylaria* sp. An isolate of *Xylaria* sp. from *Gingko biloba* had several phenolic compounds present in its methanolic extract such as 2-hydrazino-8-hydroxy-4-phenylquinoline, 3,4-dimethoxy-phenol, 2,4-bis(1,1-dimethylethyl)-phenol, 3,4-dihydro-8-hydroxy-3-methyl-isocoumarin and ferruginol. These compounds are believed to be contributors to the strong antioxidant activities exhibited by *Xylaria* sp. (Liu et al., 2007). In contrast to *Xylaria* sp., *F*. *lateritium*, did not display distinct scavenging activity. This result however is not as observed by previous studies, which regarded *Fusarium* sp. as a potent source of antioxidant based on their significant antioxidant activity recorded (Vasundhara et al., 2016). Such example include *F. oxysporum*, an endophytic fungus from the rhizomes of *Dioscorea zingiberensis* (Li et al., 2012) and *Fusarium proliferatum*, from pigeon pea (Zhao et al., 2012b).

## Data availability

The dataset generated in this study has been deposited in the GenBank, accession codes MH397067 to MH397144.

## Author contributions

TH and RM designed the study. TH performed the experiments and analyzed the data with help from SL. AH helped with antagonistic analyses. TH and SL led the writing with contributions from RT. IF-H helped in site identification and logistics. RM reviewed and edited the manuscript. All authors reviewed and approved the final manuscript.

### Conflict of interest statement

The authors declare that the research was conducted in the absence of any commercial or financial relationships that could be construed as a potential conflict of interest.

## References

[B1] Abraham S. Basukriadi A. Pawiroharsono S. Sjamsuridzal W. (2015). Insecticidal activity of ethyl acetate extracts from culture filtrates of mangrove fungal endophytes. Mycobiology 43, 137–149. 10.5941/MYCO.2015.43.2.137 26190921 PMC4505002

[B2] Aly A. H. Debbab A. Proksch P. (2011). Fungal endophytes: unique plant inhabitants with great promises. Appl. Microbiol. Biotechnol. 90, 1829–1845. 10.1007/s00253-011-3270-y 21523479

[B3] Amann R. I. Ludwig W. Schleifer K. H. (1995). Phylogenetic identification and *in situ* detection of individual microbial cells without cultivation. Microbiol. Mol. Biol. Rev. 59, 143–169. 7535888 10.1128/mr.59.1.143-169.1995 PMC239358

[B4] Amin N. (2016). Endophytic fungi to control of cocoa pod borer (*Conopomorpha cramerella*) on Cocoa plantation. Res. J. Pharm. Biol. Chem. Sci. 6:1496–1501

[B5] Anitha A. Rabeeth M. (2010). Degradation of fungal cell walls of phytopathogenic fungi by lytic enzyme of *Streptomyces griseus*. Afr. J. Plant Sci. 4, 061–066.

[B6] Bayman P. Lebron L. L. Tremblay R. L. Lodge D. J. (1997). Variation in endophytic fungi from roots and leaves of *Lepanthes* (Orchidaceae). New Phytol. 135, 143–149. 10.1046/j.1469-8137.1997.00618.x 33863156

[B7] Bernardi-Wenzel J. Garcia A. Rubim Filho C. J. Prioli A. J. Pamphile J. A. (2010). Evaluation of foliar fungal endophyte diversity and colonization of medicinal plant *Luehea divaricata* (Martius et Zuccarini). Bio. Res. 43, 375–384. 10.4067/S0716-97602010000400001 21526263

[B8] Brady S. F. Clardy J. (2000). CR377, a new pentaketide antifungal agent isolated from an endophytic fungus. J. Nat. Prod. 63, 1447–1448. 10.1021/np990568p 11076576

[B9] Cannon P. F. Simmons C. M. (2002). Diversity and host preference of leaf endophytic fungi in the Iwokrama Forest Reserve, Guyana. Mycologia 94, 210–220. 10.1080/15572536.2003.11833226 21156490

[B10] Cazar M. E. Schmeda-Hirschmann G. Astudillo L. (2005). Antimicrobial butyrolactone I derivatives from the Ecuadorian soil fungus *Aspergillus terreus* Thorn. var terreus. World J. Microbiol. Biotechnol. 21, 1067–1075. 10.1007/s11274-004-8150-526811110

[B11] Chaeprasert S. Piapukiew J. Whalley A. J. Sihanonth P. (2010). Endophytic fungi from mangrove plant species of Thailand: their antimicrobial and anticancer potentials. Bot. Mar. 53, 555–564. 10.1515/bot.2010.074

[B12] Chen J. Zhang L. Xing Y. Wang Y. Xing X. Zhang D.. (2013). Diversity and taxonomy of endophytic xylariaceous fungi from medicinal plants of *Dendrobium* (Orchidaceae). PLoS ONE 8:58268. 10.1371/journal.pone.0058268 23472167 PMC3589337

[B13] Cheng Z. Pan J. Tang W. Chen Q. Lin Y. (2009). Biodiversity and biotechnological potential of mangrove-associated fungi. J. For. Res. 20, 63–71. 10.1007/s11676-009-0012-426811110

[B14] Coşkuntuna A. Özer N. (2008). Biological control of onion basal rot disease using *Trichoderma harzianum* and induction of antifungal compounds in onion set following seed treatment. Crop Protect. 27, 330–336. 10.1016/j.cropro.2007.06.002

[B15] Crozier J. Thomas S. E. Aime M. C. Evans H. C. Holmes K. A. (2006). Molecular characterization of fungal endophytic morphospecies isolated from stems and pods of *Theobroma cacao*. Plant Pathol. 55, 783–791. 10.1111/j.1365-3059.2006.01446.x24843434

[B16] Cui J. Guo T. Ren Z. Zhang N. Wang M. (2015). Diversity and antioxidant activity of culturable endophytic fungi from alpine plants of *Rhodiola crenulata, R*. *angusta*, and *R*. sachalinensis. PLoS ONE 10:0118204. 10.1371/journal.pone.0118204 25768014 PMC4359136

[B17] da Costa Souza P. N. Grigoletto T. L. de Moraes L. A. Abreu L. M. Guimarães L. H. Santos C.. (2016). Production and chemical characterization of pigments in filamentous fungi. Microbiology 162, 12–22. 10.1099/mic.0.000168 26341482

[B18] Daouk R. K. Dagher S. M. Sattout E. J. (1995). Antifungal activity of the essential oil of *Origanum syriacum* L. J. Food Prot. 58, 1147–1149. 10.4315/0362-028X-58.10.1147 31137364

[B19] Debbab A. Aly A. H. Proksch P. (2011). Bioactive secondary metabolites from endophytes and associated marine derived fungi. Fungal Divers. 49, 1–12. 10.1007/s13225-011-0114-026811110

[B20] de Souza Sebastianes F. L. Romao-Dumaresq A. S. Lacava P. T. Harakava R. Azevedo J. L. de Melo I. S.. (2013). Species diversity of culturable endophytic fungi from Brazilian mangrove forests. Curr. Genet. 59, 153–166. 10.1007/s00294-013-0396-8 23832271

[B21] Dharmaputra O. S. (2003). Control of aflatoxigenic *Aspergillus flavus* in Peanuts Using Nonaflatoxigenic *A. flavus, A. niger* and *Trichoderma harzianum*. Biotropia 21, 32–44. 10.11598/BTB.2003.0.21.187

[B22] Douanla-Meli C. Langer E. Mouafo F. T. (2013). Fungal endophyte diversity and community patterns in healthy and yellowing leaves of *Citrus limon*. Fungal Ecol. 6, 212–222. 10.1016/j.funeco.2013.01.004

[B23] Dubey S. C. Suresh M. (2006). Randomly amplified polymorphic DNA markers for Trichoderma species and antagonism against *Fusarium oxysporum* f. *sp. ciceris* causing chickpea wilt. J. Phytopathol. 154, 663–669. 10.1111/j.1439-0434.2006.01167.x24843434

[B24] Dufossé L. Fouillaud M. Caro Y. Mapari S. A. Sutthiwong N. (2014). Filamentous fungi are large-scale producers of pigments and colorants for the food industry. Curr. Opin. Biotechnol. 26, 56–61. 10.1016/j.copbio.2013.09.007 24679259

[B25] Duke N. C. (2006). Australia's Mangroves: the Authoritative Guide to Australia's Mangrove Plants. Brisbane: University of Queensland.

[B26] Duke N. C. Meynecke J. O. Dittmann S. Ellison A. M. Anger K. Berger U.. (2007). A world without mangroves? Science 317, 41–42. 10.1126/science.317.5834.41b 17615322

[B27] Faizal A. Esyanti R. R. Aulianisa E. N. Santoso E. Turjaman M. (2017). Formation of agarwood from *Aquilaria malaccensis* in response to inoculation of local strains of *Fusarium solani*. Trees 31, 189–197. 10.1007/s00468-016-1471-926811110

[B28] Firáková S. Proksa B. Šturdíková M. (2007). Biosynthesis and biological activity of enniatins. Pharmazie 62, 563–568. 10.1691/ph.2007.8.7600 17867547

[B29] Fox E. M. Howlett B. J. (2008). Biosynthetic gene clusters for epipolythiodioxopiperazines in filamentous fungi. Mycol. Res. 112, 162–169. 10.1016/j.mycres.2007.08.017 18272357

[B30] Gaffar M. U. Morshed M. A. Uddin A. Roy S. Hannan J. M. A. (2011). Study the efficacy of *Rhizophora mucornata* Poir. leaves for diabetes therapy in Long Evans rats. Int. J. Biomol. Biomed. 1, 20–26.

[B31] Geweely N. S. (2011). Investigation of the optimum condition and antimicrobial activities of pigments from four potent pigment-producing fungal species. J. Life Sci. 5, 697–711. 10.4172/2153-2435.10000S4

[B32] Hajieghrari B. Torabi-Giglou M. Mohammadi M. R. Davari M. (2008). Biological potantial of some Iranian *Trichoderma* isolates in the control of soil borne plant pathogenic fungi. Afr. J. Biotechnol. 7, 967–972.

[B33] Hamzah T. N. T. (2018). Assessment on the Diversity and Bioactive Compounds Present in Endophytic Fungi Isolated from Rhizophora mucronata in Matang Mangrove Forest Reserve, Perak. Master's thesis, Universiti Putra Malaysia, Serdang.

[B34] Hartley S. E. Eschen R. Horwood J. M. Gange A. C. Hill E. M. (2015). Infection by a foliar endophyte elicits novel arabidopside-based reactions in its host, *Cirsium arvense*. New Phytol. 205, 816–827. 10.1111/nph.13067 25266631

[B35] He X. Han G. Lin Y. Tian X. Xiang C. Tian Q.. (2012). Diversity and decomposition potential of endophytes in leaves of a *Cinnamomum camphora* plantation in China. Ecol. Res. 27, 273–284. 10.1007/s11284-011-0898-026811110

[B36] Isaac S. (1994). Mycology answers: many fungi are brightly coloured; does pigmentation provide any advantage to those species? Mycologist 8, 178–179.

[B37] Kai M. Effmert U. Berg G. Piechulla B. (2007). Volatiles of bacterial antagonists inhibit mycelial growth of the plant pathogen *Rhizoctonia solani*. Arch. Microbiol. 187, 351–360. 10.1007/s00203-006-0199-0 17180381

[B38] Kamala T. Indira S. (2011). Evaluation of indigenous *Trichoderma* isolates from Manipur as biocontrol agent against *Pythium aphanidermatum* on common beans. 3 Biotech. 1, 217–225. 10.1007/s13205-011-0027-3 22558540 PMC3339598

[B39] Khara H. S. Hadwan H. A (1990). *In vitro* studies on antagonism of *Trichoderma* spp. against *Rhizoctonia solani* the causal agent of damping off of tomato. Plant Dis. Res. 5, 144–147.

[B40] Kjer J. Debbab A. Aly A. H. Proksch P. (2010). Methods for isolation of marine-derived endophytic fungi and their bioactive secondary products. Nat. Prot. 5, 479–490. 10.1038/nprot.2009.233 20203665

[B41] Klaiklay S. Rukachaisirikul V. Phongpaichit S. Pakawatchai C. Saithong S. Buatong J. (2012). Anthraquinone derivatives from the mangrove-derived fungus *Phomopsis* sp. PSU-MA214. Phytochem. Lett. 5, 738–742. 10.1016/j.phytol.2012.08.003

[B42] Kokpol U. Thebpatiphat S. Boonyaratavej S. Chedchuskulcai V. Ni C. Z. Clardy J. (1990). Structure of trigonostemone, a new phenanthrenone from the Thai plant *Trigonostemon reidioides*. J. Nat. Prod. 53, 1148–1151.

[B43] Koukol O. Kolarík M. Kolárová Z. Baldrian P. (2012). Diversity of foliar endophytes in wind-fallen *Picea abies* trees. Fungal Divers. 54, 69–77. 10.1007/s13225-011-0112-226811110

[B44] Kumar S. Stecher G. Tamura K. (2016). MEGA7: Molecular Evolutionary Genetics Analysis version 7.0 for bigger datasets. Mol. Biol. Evol. 33, 1870–1874. 10.1093/molbev/msw054 27004904 PMC8210823

[B45] Kusari S. Pandey S. P. Spiteller M. (2013). Untapped mutualistic paradigms linking host plant and endophytic fungal production of similar bioactive secondary metabolites. Phytochem. 91, 81–87. 10.1016/j.phytochem.2012.07.021 22954732

[B46] Landum M. C. do Rosário Félix M. Alho J. Garcia R. Cabrita M. J. Rei F.. (2016). Antagonistic activity of fungi of *Olea europaea* L. against *Colletotrichum acutatum*. Microbiol. Res. 183, 100–108. 10.1016/j.micres.2015.12.001 26805623

[B47] Lee J. C. Lobkovsky E. Pliam N. B. Strobel G. Clardy J. (1995). Subglutinols A and B: immunosuppressive compounds from the endophytic fungus *Fusarium subglutinans*. J. Org. Chem. 60, 7076–7707. 10.1021/jo00127a001

[B48] Li H. Qing C. Zhang Y. Zhao Z. (2005). Screening for endophytic fungi with antitumour and antifungal activities from Chinese medicinal plants. World J. Microbiol. Biotechnol. 21, 1515–1519. 10.1007/s11274-005-7381-4

[B49] Li P. Lou J. Mou Y. Sun W. Shan T. Zhou L. (2012). Effects of oligosaccharide elicitors from endophyitc *Fusarium oxysporum* Dzf17 on diosgenin accumulation in *Dioscorea zingiberensis* seedling cultures. J. Med. Plants Res. 6, 5128–5134. 10.5897/JMPR12.120

[B50] Liang H. Xing Y. Chen J. Zhang D. Guo S. Wang C. (2012). Antimicrobial activities of endophytic fungi isolated from *Ophiopogon japonicus* (Liliaceae). BMC Compl. Alt. Med. 12:238. 10.1186/1472-6882-12-238 23190550 PMC3534486

[B51] Liu X. Dong M. Chen X. Jiang M. Lv X. Yan G. (2007). Antioxidant activity and phenolics of an endophytic *Xylaria* sp. from *Ginkgo biloba*. Food Chem. 105, 548–554. 10.1016/j.foodchem.2007.04.008

[B52] Liu X. Dong M. Chen X. Jiang M. Lv X. Zhou J. (2008). Antimicrobial activity of an endophytic *Xylaria* sp. YX-28 and identification of its antimicrobial compound 7-amino-4-methylcoumarin. Appl. Microbiol. Biotechnol. 78, 241–247. 10.1007/s00253-007-1305-1 18092158

[B53] Maharachchikumbura S. S. Guo L. D. Cai L. Chukeatirote E. Wu W. P. Sun X.. (2012). A multi-locus backbone tree for *Pestalotiopsis*, with a polyphasic characterization of 14 new species. Fungal Divers. 56, 95–129. 10.1007/s13225-012-0198-126811110

[B54] Mapari S. A. Thrane U. Meyer A. S. (2010). Fungal polyketide azaphilone pigments as future natural food colorants? Trends Biotechnol. 28, 300–307. 10.1016/j.tibtech.2010.03.004 20452692

[B55] Oliveira A. L. L. D. Felício R. D. Debonsi H. M. (2012). Marine natural products: chemical and biological potential of seaweeds and their endophytic fungi. Braz. J. Pharm. 22, 906–920. 10.1590/S0102-695X2012005000083

[B56] Onn M. L. Lim P. T. Mujahid A. Proksch P. Müller M. (2016). Initial screening of mangrove endophytic fungi for antimicrobial compounds and heavy metal biosorption potential. Sains Malaysiana, 45, 1063–1071.

[B57] Park J. H. Choi G. J. Lee H. B. Kim K. M. Jung H. S. Lee S. W. (2005). Griseofulvin from *Xylaria* sp. strain F0010, an endophytic fungus of *Abies holophylla* and its antifungal activity against plant pathogenic fungi. J. Microbiol. Biotechnol. 15, 112–117.

[B58] Phongpaichit S. Rungjindamai N. Rukachaisirikul V. Sakayaroj J. (2006). Antimicrobial activity in cultures of endophytic fungi isolated from *Garcinia* species. FEMS Immun. Med. Microbiol. 48, 367–372. 10.1111/j.1574-695X.2006.00155.x 17052267

[B59] The Plant List (2013). Version 1.1. Published on the Internet. Available Online at: http://www.theplantlist.org/

[B60] Polidoro B. A. Carpenter K. E. Collins L. Duke N. C. Ellison A. M. Ellison J. C.. (2010). The loss of species: mangrove extinction risk and geographic areas of global concern. PLoS ONE 5:e10095. 10.1371/journal.pone.0010095 20386710 PMC2851656

[B61] Potshangbam M. Devi S. I. Sahoo D. Strobel G. A. (2017). Functional characterization of endophytic fungal community associated with *Oryza sativa* L. and *Zea mays* L. Front. Microbiol. 8:325. 10.3389/fmicb.2017.00325 28303127 PMC5332368

[B62] Premalatha B. Pradeep F. S. Pradeep B. V. Palaniswamy M. (2012). Production and characterization of naphthoquinone pigment from *Fusarium moniliforme* MTCC6985. World. J. Pharm. Res. 1, 1126–1142.

[B63] Prihanto A. A. Firdaus M. Nurdiani R. (2011). Endophytic fungi isolated from mangrove (*Rhyzopora mucronata*) and its antibacterial activity on *Staphylococcus aureus* and *Escherichia coli*. J. Food Sci. Eng. 1, 386–389.

[B64] Qin H. Boufford D. E. (2007). Rhizophoraceae, in Flora of China, Vol. 13, eds Wu Z. Raven P. H. Hong D. (Beijing; St. Louis, MO: Science Press; Missouri Botanical Garden Press), 295–299.

[B65] Ramasamy K. Lim S. M. Bakar H. A. Ismail N. Ismail M. S. Ali M. F.. (2010). Antimicrobial and cytotoxic activities of Malaysian endophytes. Phytoth. Res. 24, 640–643. 10.1002/ptr.2891 19468989

[B66] Ratnaweera P. B. de Silva E. D. Williams D. E. Andersen R. J. (2015). Antimicrobial activities of endophytic fungi obtained from the arid zone invasive plant *Opuntia dillenii* and the isolation of equisetin, from endophytic *Fusarium sp*. BMC Compl. Alt. Med. 15:220. 10.1186/s12906-015-0722-4 26160390 PMC4496918

[B67] dos Reis Celestino J. de Carvalho L. E. da Paz Lima M. Lima A. M. Ogusku M. M. de Souza J. V. B. (2014). Bioprospecting of Amazon soil fungi with the potential for pigment production. Proc. Biochem. 49, 569–575. 10.1016/j.procbio.2014.01.018

[B68] Rhoden S. A. Garcia A. Bongiorno V. A. Azevedo J. L. Pamphile J. A. (2012). Antimicrobial activity of crude extracts of endophytic fungi isolated from medicinal plant *Trichilia elegans* A. Juss. J. App. Pharm. Sci. 02, 57–59. 10.7324/JAPS.2012.2807

[B69] Robinson J. G. Nedergaard B. S. Rogers W. J. Fialkow J. Neutel J. M. Ramstad D.. (2014). Effect of evolocumab or ezetimibe added to moderate-or high-intensity statin therapy on LDL-C lowering in patients with hypercholesterolemia: the LAPLACE-2 randomized clinical trial. JAMA. 311, 1870–1883. 10.1001/jama.2014.4030 24825642

[B70] Rodriguez R. J. Henson J. Van V. Hoy M. Wright L. Beckwith F.. (2008). Stress tolerance in plants via habitat-adapted symbiosis. Int. Soc. Microbiol. Ecol. J. 2, 404–416. 10.1038/ismej.2007.106 18256707

[B71] Score A. J. Palfreyman J. W. White N. A. (1997). Extracellular phenoloxidase and peroxidase enzyme production during interspecific fungal interactions. Int. Biodeter. Biodegrad. 39, 225–233.

[B72] Setyawan A. D. Ulumuddin Y. I. (2012). Species diversity of *Rhizophora* in Tambelan Islands, Natuna Sea, Indonesia. Biodiversitas 13, 172–177. 10.13057/biodiv/d130402

[B73] Sharon E. Bar-Eyal M. Chet I. Herrera-Estrella A. Kleifeld O. Spiegel Y. (2001). Biological control of the root-knot nematode *Meloidogyne javanica* by *Trichoderma harzianum*. Phytopathol. 91, 687–693. 10.1016/j.soilbio.2008.03.011 18942999

[B74] Shreelalitha S. J. Sridhar K. R. (2015). Endophytic fungi of wild legume *Sesbania bispinosa* in coastal sand dunes and mangroves of the Southwest coast of India. J. For. Res. 26, 1003–1011. 10.1007/s11676-015-0103-326811110

[B75] Siameto E. N. Okoth S. Amugune N. O. Chege N. C. (2010). Antagonism of *Trichoderma farzianum* isolates on soil borne plant pathogenic fungi from Embu District, Kenya. J. Yeast Fungal Res. 1, 47–54.

[B76] Singh S. K. Srivastava P. K. Gupta M. Thakur J. K. Mukherjee S. (2014). Appraisal of land use/land cover of mangrove forest ecosystem using support vector machine. Env. Earth Sci. 71, 2245–2255. 10.1007/s12665-013-2628-026811110

[B77] Suganthy N. Pandima Devi K. (2016). *In vitro* antioxidant and anti-cholinesterase activities of *Rhizophora mucronata*. Pharm. Biol. 54, 118–129. 10.3109/13880209.2015.1017886 25856713

[B78] Suryanarayanan T. S. Murali T. S. Venkatesan G. (2002). Occurrence and distribution of fungal endophytes in tropical forests across a rainfall gradient. Can. J. Bot. 80, 818–826. 10.1139/b02-069

[B79] Teixeira M. F. Martins M. S. Da Silva J. C. Kirsch L. S. Fernandes O. C. Carneiro A. L. (2012). Amazonian biodiversity: pigments from *Aspergillus* and *Penicillium*-characterizations, antibacterial activities and their toxicities. Curr. Trends in Biotech. Pharm. 6, 300–311.

[B80] Thomas S. A. L. Fleming R. Shaw L. N. Baker B. J. (2016). Isolation of bioactive secondary metabolites from mangrove fungal endophytes using epigenetic regulation. Planta Med. 82, S1–S381. 10.1055/s-0036-159673726606158

[B81] Tomlinson P. B. (1986). The Botany of Mangroves. Cambridge Tropical Biology Series. New York, NY: Cambridge University Press.

[B82] Tudor D. Robinson S. C. Cooper P. A. (2013). The influence of pH on pigment formation by lignicolous fungi. Int. Biodet. Biodegrad. 80, 22–28. 10.1016/j.ibiod.2012.09.013

[B83] Udayanga D. Liu X. McKenzie E. H. Chukeatirote E. Bahkali A. H. Hyde K. D. (2011). The genus *Phomopsis*: biology, applications, species concepts and names of common phytopathogens. Fungal Divers. 50, 189–225. 10.1007/s13225-011-0126-926811110

[B84] Umechuruba C. I. (2005). Health impact assessment of mangrove vegetation in an oil spilled site at the Bodo West field in Rivers State, Nigeria. J. Appl. Sci. Environ. Manag. 9, 69–73.

[B85] Vasundhara M. Baranwal M. Kumar A. (2016). *Fusarium tricinctum*, an endophytic fungus exhibits cell growth inhibition and antioxidant activity. Ind. J. Microbiol. 56, 433–438. 10.1007/s12088-016-0600-x 27784939 PMC5061693

[B86] Wang Q. Li S. Zhao F. Dai H. Bao L. Ding R.. (2011). Chemical constituents from endophytic fungus *Fusarium oxysporum*. Fitoterapia 85, 777–781. 10.1016/j.fitote.2011.04.002 21497643

[B87] Wheeler K. A. Hocking A. D. (1993). Interactions among xerophilic fungi associated with dried salted fish. J. Appl. Microbiol. 74, 164–169. 8444646 10.1111/j.1365-2672.1993.tb03010.x

[B88] White T. J. Bruns T. Lee S. J. W. T. Taylor J. L. (1990). Amplification and direct sequencing of fungal ribosomal RNA genes for phylogenetics. PCR protocols: a guide to methods and applications 18, 315–322. 10.1016/B978-0-12-372180-8.50042-1

[B89] Zhao J. Fu Y. Luo M. Zu Y. Wang W. Zhao C.. (2012a). Endophytic fungi from pigeon pea [*Cajanus cajan* (L.) Millsp.] produce antioxidant cajaninstilbene acid. J. Agr. Food Chem. 60, 4314–4319. 10.1021/jf205097y 22494407

[B90] Zhao Y. Wei T. Yin K. Chen Z. Gu H. Qu L.. (2012b). Arabidopsis RAP2. 2 plays an important role in plant resistance to *Botrytis cinerea* and ethylene responses. New Phytologist 195, 450–460. 10.1111/j.1469-8137.2012.04160.x 22530619

