# Supplementary material for: Diversity and Characterization of Endophytic Fungi Isolated From the Tropical Mangrove Species, Rhizophora mucronata, and Identification of Potential Antagonists Against the Soil-Borne Fungus, Fusarium solani
Source: Front Microbiol. 2018 Jul 25;9:1707. doi: 10.3389/fmicb.2018.01707 (PMC6068387; doi:10.3389/fmicb.2018.01707)
Supplement: Supplementary file 1 [file Table_1.doc]

**Diversity and characterization of endophytic fungi isolated from the tropical mangrove species, *Rhizophora mucronata*, and identification of potential antagonists against the soil-borne fungus, *Fusarium solani***

Tuan Noraida Tuan HAMZAH, Shiou Yih LEE, Asep HIDAYAT, Razak TERHEM, Ibrahim FARIDAH-HANUM, Rozi MOHAMED*,

Supplementary Table S1 Morphological characteristics of 350 endophytic fungi isolated from *Rhizophora mucronata* leaves

| Colony colour | Group | Morphology Characteristics | | | Isolate ID |
| --- | --- | --- | --- | --- | --- |
|  |  | Texture | Surface | Opacity |  |
| White | A.01 | Floccose, woolly | White, smooth, filamentous colony form, edge of colony entire | Opaque | RM1.18A.20, RM1.18A.54, RM3.18A.06, RM3.18A.67, RM2.30.11, RM2.30.19 |
| White | A.02 | Velvety | White, smooth, filamentous colony form, abundant growth, edge of colony entire | Opaque | RM2.18A.15, RM2.18A.51, RM2.18A.54, RM3.18A.22, RM3.18A.43, RM3.18A.57 |
| White | A.03 | Floccose, velvety | White, concentric, undulate colony form, cotton-like mycelia, edge of colony entire | Opaque | RM1.18A.01, RM1.18A.03, RM1.18A.44, RM1.18A.52, RM1.18A.60, RM2.18A.19, RM2.18A.21, RM2.18A.27, RM2.18A.35, RM3.18A.04, RM3.18A.40, RM3.18A.35, RM3.18A.38 |
| White | A.04 | Floccose, Powdery | Off white, wooly, filamentous colony form, edge of colony filiform | Opaque | RM1.30.17, RM1.30.45,  RM1.30.49, RM2.30.04,  RM2.30.13, RM2.30.27,  RM3.30.42, RM3.30.51 |
| White | A.05 | Floccose, dry | White mycelia, wooly, filamentous colony form, moderate amount of mycelia, edge of colony filiform | Opaque, some areas translucent | RM1.18A.02, RM1.18A.17, RM1.18A.27, RM1.18A.39, RM1.18A.57, RM2.18A.11, RM2.18A.24, RM3.18A.07, RM3.18A.59, RM1.30.29, RM1.30.48, RM1.30.50, RM2.30.22, RM2.30.43, RM3.30.01 |
| hite | A.06 | Powdery, Dry | White mycelia, circular colony form, heavy presence of sclerotia, abundant growth of mycelia, edge of colony filiform | Opaque | RM1.18A.11, RM1.18A.23, RM1.18A.30, RM1.18A.37, RM2.18A.25, RM3.18A.03, RM3.18A.34, RM3.18A.39, RM3.18A.50, RM3.18A.51, RM3.18A.62, RM1.30.11, RM1.30.33, RM1.30.46, RM1.30.47 |
| White | A.07 | Powdery, dry | White mycelia, poor presence of sclerotia, filamentous colony form, moderate growth of mycelia, edge of colony filiform | Opaque, translucent | RM1.30.12, RM1.30.13, RM1.30.39, RM1.30.53, RM2.30.44, RM2.30.45, RM3.30.38 |
| White | A.08 | Smooth, powdery | White mycelium, grayish at the center of the colony, abundant growth, filamentous colony form, edge of colony filiform | Opaque | RM1.18A.13, RM1.18A.16, RM1.18A.33, RM1.18A.38, RM1.18A.47, RM2.18A.12, RM2.18A.13, RM2.18A.23, RM2.18A.30, RM2.18A.44 |
| White | A.09 | Floccose, dry | White, concentric at the center of colony, filamentous colony form, edge of colony lobate | Opaque at center, translucent | RM1.18A.06, RM1.18A.41, RM1.18A.45, RM1.18A.48 |
| White | A.10 | Smooth, fluffy | White, cotton-like mycelia, abundant growth, irregular colony form, edge of colony undulate | Opaque | RM1.30.05, RM1.30.36, RM1.30.37, RM3.30.50, RM3.30.54, RM3.30.57 |
| White | A.11 | Floccose, dry | White, flower-like mycelial growth development, irregular colony form, edge of colony filiform | Opaque | RM2.30.03, RM2.30.17, RM2.30.29, RM2.30.36, RM2.30.37, RM2.30.49, RM2.30.50 |
| White | A.12 | Floccose, woolly, dry | White, cotton-like, filamentous colony form, edge of colony undulate | Opaque | RM2.18A.06, RM2.18A.08, RM2.18A.09, RM2.18A.16, RM2.18A.17, RM2.18A.42, RM2.18A.47, RM2.18A.49, RM2.18A.53 |
| White | A.13 | Floccose, dry | White, filamentous colony form, moderate growth of mycelia, flower like mycelia growth development, edge of colony undulate | Opaque, translucent | RM1.30.31, RM1.30.32, RM2.30.09, RM2.30.21, RM2.30.47, RM3.30.11, RM3.30.23 |
| White | A.14 | Floccose, velvety | White, filamentous colony form, abundant growth of mycelia, edge of colony undulate, filiform | Opaque | RM2.18A.01, RM2.18A.10, RM2.18A.34, RM2.18A.37, RM2.18A.45 |
| White | A.15 | Floccose, velvety | White, cotton-like mycelia, circular colony form, abundant growth of mycelia, edge of colony curled | Opaque | RM3.18A.01, RM3.18A.02, RM3.18A.29, RM3.18A.30, RM3.18A.33, RM3.18A.47, RM3.18A.52, RM3.18A.66 |
| White | A.16 | Powdery, dry | White, filamentous colony form, abundant growth of mycelia, edge of colony filiform | Opaque | RM2.18A.02, RM2.18A.03, RM2.18A.20, RM2.18A.39, RM2.18A.43, RM2.18A.55 |
| White | A.17 | Powdery | White, black concentric mycelia at the center of the colony, filamentous colony form, abundant growth, edge of colony filiform | Opaque | RM3.18A.11, RM3.18A.46, RM3.18A.55, RM3.18A.63 |
| White | A.18 | Smooth, glistening | White, white to light yellowish mycelia, circular colony form, abundant growth, edge of colony entire | Opaque | RM1.30.02, RM1.30.08, RM1.30.09, RM1.30.27, RM1.30.44 |
| White | A.19 | Powdery, dry | White, circular ring black mycelia at the center of the colony, irregular colony form, abundant growth, edge of colony undulate | Opaque | RM3.30.06, RM3.30.31, RM3.30.36 |
| White | A.20 | Powdery, dry | White, dark grey circular ring at center of the colony, filamentous colony from, edge of colony filiform | Opaque | RM3.18A.25, RM3.18A.44, RM3.18A.60 |
| Green | B.01 | Floccose, smooth, fluffy | Soft olive green, circular layer with various shades of green, black green, chrome green, circular colony form, abundant growth, edge of colony entire | Opaque | RM3.30.08, RM3.30.27, RM3.30.32, RM3.30.33, RM3.30.41 |
| Green | B.02 | Floccose, smooth, fluffy | Reed green, circular colony form, abundant growth, edge of colony entire | Opaque | RM1.18A.04, RM1.18A.18, RM1.18A.36, RM1.18A.55. RM1.18A.61 |
| Green | B.03 | Floccose, fluffy | Dark olive green, circular layer black green, circular colony form, abundant growth, edge of colony entire | Opaque | RM1.18A.09, RM1.18A.19, RM1.18A.35, RM1.18A.43, RM1.18A.46, RM1.18A.51 |
| Green | B.04 | Floccose, dry | Yellow green, circular layer at the center of colony black green, outer layer white, irregular colony form, moderate growth, edge of colony undulate | Opaque | RM1.30.03, RM1.30.23, RM1.30.24, RM1.30.35 |
| Green | B.05 | Powdery, dry | Dark olive green, layers from the center of colony ranging from cream, black green, outer layer off white, circular colony form, abundant growth, edge of colony entire | Opaque | RM3.18A.20, RM3.18A.36, RM3.18A.49 |
| Green | B.06 | Powdery, dry | Olive green, outer layer white, circular colony form, abundant growth, edge of colony entire-filiform | Opaque | RM2.30.01, RM2.30.30, RM2.30.31 |
| Green | B.07 | Fluffy, smooth | Soft green-brown, cotton-like mycelia, circular colony form, abundant growth, edge of colony entire | Opaque | RM3.30.09, RM3.30.44, RM3.30.48 |
| Green | B.08 | Floccose | Dark olive green, circular colony form, abundant growth, edge of colony entire | Opaque | RM1.30.04, RM1.30.19, RM1.30.25, RM2.30.23, RM3.30.30, RM3.30.34 |
| Green | B.09 | Fluffy, smooth | Reed green, circular colony form, abundant growth, edge of colony, entire | Opaque | RM.1.30.10, RM1.30.41, RM2.30.32, RM3.30.37 |
| Green | B.10 | Floccose, woolly | Black green, white mycelia, filamentous colony form, abundant growth, edge of colony filiform | Opaque | RM3.30.04, RM3.30.24, RM3.30.26, RM3.30.47 |
| Green | B.11 | Powdery, floccose, dry | Light brown green, creamy circular layer, irregular colony form, abundant growth, edge of colony undulate | Opaque | RM1.30.01, RM1.30.18, RM1.30.40, RM1.30.52 |
| Green | B.12 | Smooth, velvety, glistening | Emerald green, circular colony form, abundant growth, edge of colony entire | Opaque | RM3.18A.08, RM3.18A.31, RM3.18A.54 |
| Green | B.13 | Floccose, dry | Black green, light olive green mycelia, circular colony form, abundant growth, edge of colony entire | Opaque | RM2.30.07, RM2.30.33 |
| Green | B.14 | Floccose | Fern green, creamy outer layer, circular colony form, moderate growth, edge of colony entire | Opaque | RM3.18A.23, RM3.18A.42 |
| Green | B.15 | Rough | Black green, heavy sclerotia present, irregular colony form, abundant growth, edge of colony undulate | Opaque | RM3.18A.17, RM3.18A.41 |
| Green | B.16 | Floccose, woolly | Blue green, creamy woolly outer layer, filamentous colony form, abundant growth, edge of colony filiform | Opaque | RM3.18A.21 |
| Green | B.17 | Powdery, dry | Blue green, creamy outer layer, circular colony form, abundant growth, edge of colony entire | Opaque | RM2.30.06, RM2.30.25, RM2.30.51 |
| Green | B.18 | Floccose, dry | Olive green, creamy and dark green outer layer, circular colony form, abundant growth, edge of colony filiform | Opaque | RM2.30.15, RM2.30.35 |
| Green | B.19 | Fluffy, smooth | Dark green, white outer layer, circular colony form, abundant growth, edge of colony entire | Opaque | RM1.30.26, RM1.30.43, RM1.30.51, RM2.30.12, RM3.30.07 |
| Green | B.20 | Fluffy, smooth | Light green, light brown and creamy outer layer, circular colony form, abundant growth, edge of colony entire | Opaque | RM3.30.02, RM3.30.15, RM3.30.20 |
| Grey | C.01 | Floccose, smooth | Light grey, creamy outer layer, circular colony form, abundant growth, edge of colony filiform | Opaque | RM3.18A.10 |
| Grey | C.02 | Floccose, woolly | Gray, creamy outer layer, irregular colony form, abundant growth, edge of colony undulate | Opaque | RM2.18A.18, RM2.18A.33, RM3.18A.15 |
| Grey | C.03 | Floccose, woolly | Gray, light brown and brown outer layer, circular colony form, moderate growth, edge of colony filiform | Opaque | RM3.18A.14, RM3.18A.45 |
| Grey | C.04 | Floccose, woolly | White-grayish, irregular colony growth, abundant growth, edge of colony filiform | Opaque | RM1.30.06, RM1.30.30, RM1.30.42, RM.2.30.14, RM2.30.39, RM2.30.40 |
| Grey | C.05 | Floccose, woolly | Light grey, brown circular ring, circular colony form, abundant growth, edge of colony filiform | Opaque | RM3.30.19, RM3.30.46 |
| Grey | C.06 | Floccose, rough | Green-grayish, light brown and dark brown outer layer, concentric white mycelium at the center of colony, irregular colony form, abundant growth, edge of colony filiform | Opaque | RM1.30.15, RM1.30.20, RM1.30.28, RM1.30.34, RM1.30.38, RM3.30.16, RM3.30.49 |
| Grey | C.07 | Floccose, woolly | Dim grey, concentric growth, irregular colony form, abundant growth, edge of colony undulate, produced violet pigmentation into medium | Opaque | RM2.30.06, RM2.30.34, RM2.30.46 |
| Grey | C.08 | Floccose, smooth | Dim grey, circular colony form, abundant growth, edge of colony entire | Opaque | RM3.18A.24, RM2.18A.38 |
| Grey | C.09 | Floccose, woolly | Dark grey, white outer layer, circular colony form, abundant growth, edge of colony filiform | Opaque | RM3.18A.18, RM3.18A.58 |
| Grey | C.10 | Floccose, woolly | Yellow grey, circular colony form, abundant growth, edge of colony filiform | Opaque | RM3.30.22, RM3.30.40 |
| Black | C.11 | Floccose, woolly | Black, covered with white mycelium on the upper part, circular colony form, abundant growth, edge of colony filiform | Opaque | RM3.30.21, RM3.30.55 |
| Black | C.12 | Floccose, woolly | Black, circular colony form, abundant growth, edge of colony filiform | Opaque | RM1.18A.14, RM1.18A.22, RM1.18A.25, RM1.18A.34, RM1.18A.50, RM1.18A.63 |
| Grey | C.13 | Powdery, floccose | Black, white outer layer, circular colony form, abundant growth, edge of colony filiform | Opaque | RM3.30.14, RM3.30.28, RM3.30.56 |
| Brown | D.01 | Fluffy, smooth | Light brown, circular colony form, abundant growth, edge of colony entire | Opaque | RM1.18A.59, RM2.18A.05, RM2.18A.31, RM2.18A.48, RM2.18A.56, RM2.18A.57 |
| Brown | D.02 | Fluffy, smooth | Creamy-light brown, irregular colony form, abundant growth, edge of colony undulate | Opaque | RM3.30.03, RM3.30.35 |
| Brown | D.03 | Fluffy, velvety, smooth | Cream-brownish, circular colony form, abundant growth, edge of colony filiform | Opaque | RM3.30.05, RM3.30.43, RM3.30.45 |
| Brown | D.04 | Floccose, woolly | Brown, irregular colony form, abundant growth, edge of colony undulate | Opaque | RM1.18A.58, RM2.18A.26, RM2.18A.40, RM3.18A.19 |
| Brown | D.05 | Floccose | Soft brown, dark brown at the center of the colony, irregular colony growth, abundant growth, edge of colony undulate | Opaque | RM3.30.10, RM3.30.25, RM3.30.53 |
| Brown | D.06 | Fluffy, smooth | Soft beige-brown, circular colony form, abundant growth, edge of colony entire | Opaque | RM3.18A.13, RM3.18A.44, RM3.18A.64 |
| Brown | D.07 | Fluffy | Light brown, concentric cotton-like white mycelia at the center of the colony, abundant growth, edge of colony filiform | Opaque | RM1.30.14, RM2.30.08, RM2.30.09, RM2.30.26, RM2.30.48 |
| Brown | D.08 | Fluffy | Soft beige brown, concentric light green mycelia at the center of colony, cotton like mycelia, circular colony form, abundant growth, edge of colony entire | Opaque | RM2.18A.28, RM2.18A.41, RM3.18A.09, RM3.18A.32, RM3.18A.56, RM3.18A.61 |
| Brown | D.09 | Floccose | Light brown, concentric white mycelia at the center of the colony, circular colony form, abundant growth, edge of colony entire | Opaque | RM1.18A.12 RM1.18A.31 RM1.18A.58 RM2.18A.46 |
| Brown | D.10 | Fluffy, woolly | Beige-brown, circular colony form, abundant growth, edge of colony filiform-entire | Opaque | RM2.30.10, RM2.30.16, RM2.30.24, RM2.30.38, RM2.30.52, RM2.30.54 |
| Brown | D.11 | Fluffy, floccose | Beige-light brown, circular concentric mycelium at the center of the colony, circular colony form, filamentous outer layer, abundant growth, edge of colony filiform | Opaque | RM1.30.07, RM1.30.16, RM1.30.21, RM1.30.22 |
| Brown | D.12 | Floccose, powdery | Brown, several layers with several colors, dark brown for the most outer layer, brown for next layer, light brown towards the middle of colony, concentric brown mycelia at the center of colony, irregular colony growth, abundant growth, edge of colony undulate | Opaque | RM2.30.05, RM2.30.20, RM2.30.42 |
| Brown | D.13 | Floccose, woolly | Brown, dark brown at the most outer layer, followed by green brown, concentric flower-like dark olive green mycelium at the center of colony, irregular colony form, abundant growth, edge of colony filiform | Opaque | RM3.18A.26, RM3.18A.28, RM3.18A.37 |
| Brown | D.14 | Floccose | Light brown, concentric dark olive green mycelia at the center of colony, irregular colony growth, abundant growth, edge of colony undulate | Opaque | RM3.18A.16, RM3.18A.27, RM3.18A.65 |
| Brown | D.15 | Floccose, woolly | Brown-peach, cotton-like mycelia, circular colony form, abundant growth, edge of colony filiform | Opaque | RM2.18A.04, RM2.18A.22, RM2.18A.36, RM2.18A.50, RM2.18A.52 |
| Orange | E.01 | Powdery, dry | Orange, irregula colony form, abundant growth, edge of colony undulate | Opaque | RM1.18A.05, RM1.18A.28, RM1.18A.29, RM1.18A.42, RM2.18A.32, RM2.30.02, RM2.30.41, RM2.30.53 |
| Orange | E.02 | Powdery, dry | Light orange, irregular colony form, abundant growth, edge of colony undulate | Opaque | RM1.18A.07, RM1.18A.21, RM1.18A.26, RM1.18A.62 |
| Orange | E.03 | Floccose, powdery | Orange, rhizoid colony form, abundant growth, edge of colony undulate | Opaque | RM3.18A.05, RM3.18A.53 |
| Orange | E.04 | Powdery | Light-orange, irregular colony form, abundant growth, edge of colony undulate | Opaque | RM3.30.13, RM3.30.17, RM3.30.39, RM3.30.52 |
| Yellow | E.05 | Floccose, glistening, velvety | Yellowish-white shimmering, filamentous colony form, abundant growth, edge of colony filiform | Opaque | RM1.18A.08, RM1.18A.32, RM1.18A.49, RM1.18A.53 |
| Purple | E.06 | Smooth | Purple, circular colony form, moderate growth, edge of colony entire | Transparent | RM3.30.12, RM3.30.29 |
| Violet | E.07 | Floccose, woolly | Violet-purple-yellowish, circular colony form, abundant growth, edge of colony entire | Opaque | RM1.18A.10, RM1.18A.15, RM1.18A.24, RM1.18A.40 |
| Purple | E.08 | Floccose | Purple, filamentous colony form, moderate growth, edge of colony filiform | Translucent | RM2.18A.07, RM2.18A.14, RM2.18A.29 |
