# Supplementary material for: Diversity and Characterization of Endophytic Fungi Isolated From the Tropical Mangrove Species, Rhizophora mucronata, and Identification of Potential Antagonists Against the Soil-Borne Fungus, Fusarium solani
Source: Front Microbiol. 2018 Jul 25;9:1707. doi: 10.3389/fmicb.2018.01707 (PMC6068387; doi:10.3389/fmicb.2018.01707)
Supplement: Supplementary file 2 [file Table_2.doc]

**Diversity and characterization of endophytic fungi isolated from the tropical mangrove species, *Rhizophora mucronata*, and identification of potential antagonists against the soil-borne fungus, *Fusarium solani***

Tuan Noraida Tuan HAMZAH, Shiou Yih LEE, Asep HIDAYAT, Razak TERHEM, Ibrahim FARIDAH-HANUM, Rozi MOHAMED*,

Supplementary Table S2 GenBank accessions downloaded as reference species for Maximum Likelihood tree construction using the ITS sequence

| No. | Species | GenBank Accession |
| --- | --- | --- |
| 1 | *Alternaria agerati* | KJ718098 |
| 2 | *Alternaria alternata* | KY609180 |
| 3 | *Alternaria arborescens* | KP942903 |
| 4 | *Alternaria brassicicola* | KF542552 |
| 5 | *Alternaria dauci* | AY154701 |
| 6 | *Alternaria eichhorniae* | KU593527 |
| 7 | *Alternaria longipes* | KJ722535 |
| 8 | *Alternaria macrospora* | AY154689 |
| 9 | *Alternaria porri* | KX139162 |
| 10 | *Alternaria sesami* | JF780939 |
| 11 | *Alternaria sesamicola* | AF314588 |
| 12 | *Alternaria steviae* | MF471694 |
| 13 | *Alternaria solani* | KT721914 |
| 14 | *Alternaria tenuissima* | KY859406 |
| 15 | *Alternaria tillandsiae* | KU144925 |
| 16 | *Arthrinium urticae* | AB220245 |
| 17 | *Arthrinium* sp. | HQ022507 |
| 18 | *Auriculariopsis ampla* | L43382 |
| 19 | *Cladosporium asperulatum* | LN834357 |
| 20 | *Cladosporium chlorocephalum* | AF393686 |
| 21 | *Cladosporium cladosporioides* | KM980007 |
| 22 | *Cladosporium colocasiae* | AF393692 |
| 23 | *Cladosporium globisporum* | KP701967 |
| 24 | *Cladosporium oxysporum* | AF393720 |
| 25 | *Cladosporium perangustum* | KP701968 |
| 26 | *Cladosporium tenuissimum* | LN834397 |
| 27 | *Curvularia aeria* | KT933642 |
| 28 | *Curvularia affinis* | HG778982 |
| 29 | *Curvularia brachyspora* | HG778983 |
| 30 | *Curvularia chlamydospora* | HG779022 |
| 31 | *Curvularia clavata* | KU715109 |
| 32 | *Curvularia geniculata* | KM979949 |
| 33 | *Curvularia lunata* | DQ836800 |
| 34 | *Curvularia prasadii* | HG778996 |
| 35 | *Curvularia senegalensis* | HG779001 |
| 36 | *Diaporthe arecae* | KC343032 |
| 37 | *Diaporthe discoidispora* | KY011887 |
| 38 | *Diaporthe helianthi* | KM979906 |
| 39 | *Diaporthe lithocarpus* | KR703276 |
| 40 | *Diaporthe longicolla* | KX977489 |
| 41 | *Diaporthe phaseolorum* | EF488425 |
| 42 | *Diaporthe sackstonii* | KJ197287 |
| 43 | *Diaporthe sojae* | KX355197 |
| 44 | *Diaporthe thunbergiicola* | KP715097 |
| 45 | *Diaporthe unshiuensis* | MF185130 |
| 46 | *Diaporthe yunnanensis* | KY491542 |
| 47 | *Epicoccum nigrum* | KR094452 |
| 48 | *Fusarium equiseti* | KY365254 |
| 49 | *Fusarium fujikuroi* | KX674663 |
| 50 | *Fusarium lateritium* | AY266406 |
| 51 | *Fusarium oxysporum* | KJ605154 |
| 52 | *Fusarium proliferatum* | KY039293 |
| 53 | *Fusarium sarcochroum* | LT746257 |
| 54 | *Fusarium verticillioides* | KR183784 |
| 55 | *Leptosphaeria microscopica* | MF576268 |
| 56 | *Leptosphaerulina chartarum* | KJ398148 |
| 57 | *Leptosphaeria* sp. | KM979814 |
| 58 | *Letendraea helminthicola* | [KP263123](https://www.ncbi.nlm.nih.gov/nucleotide/KP263123.1?report=genbank&log$=nucltop&blast_rank=2&RID=6KAUTY4G015) |
| 59 | *Letendraea helminthicola* | [KU529827](https://www.ncbi.nlm.nih.gov/nucleotide/KU529827.1?report=genbank&log$=nucltop&blast_rank=1&RID=6KAUTY4G015) |
| 60 | *Lophiostoma cordylinicola* | KM213996 |
| 61 | *Lophiostoma helminthicola* | JQ026217 |
| 62 | *Lophiostoma helminthicola* | JN116664 |
| 63 | *Neopestalotiopsis australis* | KY398730 |
| 64 | *Neopestalotiopsis clavispora* | KY319134 |
| 65 | *Neopestalotiopsis egyptiaca* | KP943747 |
| 66 | *Neopestalotiopsis eucalypticola* | KM199376 |
| 67 | *Neopestalotiopsis foedans* | KU593530 |
| 68 | *Neopestalotiopsis formicarum* | KM199344 |
| 69 | *Neopestalotiopsis mesopotamica* | KM199362 |
| 70 | *Neopestalotiopsis piceana* | KM199372 |
| 71 | *Neopestalotiopsis protearum* | KT936425 |
| 72 | *Neopestalotiopsis saprophytica* | KY082708 |
| 73 | *Nigrospora oryzae* | KM979813 |
| 74 | *Nigrospora pyriformis* | KX985940 |
| 75 | *Nigrospora sphaerica* | KM510416 |
| 76 | *Paraphaeosphaeria angularis* | JX496047 |
| 77 | *Paraphaeosphaeria michotii* | JX496079 |
| 78 | *Paraphaeosphaeria sporulosa* | KT184776 |
| 79 | *Paraphaeosphaeria verruculosa* | JX496059 |
| 80 | *Paraphaeosphaeria* sp. | KX611058 |
| 81 | *Periconia aquatica* | KY794701 |
| 82 | *Periconia byssoides* | KC954157 |
| 83 | *Periconia pseudobyssoides* | KC954161 |
| 84 | *Pestalotiopsis maculans* | KX610327 |
| 85 | *Pestalotiopsis gracilis* | AF409962 |
| 86 | *Pestalotiopsis malicola* | JF501649 |
| 87 | *Pestalotiopsis microspora* | FJ478120 |
| 88 | *Pestalotiopsis paeoniicola* | FJ997645 |
| 89 | *Pestalotiopsis protearum* | JX556231 |
| 90 | *Pestalotiopsis sydowiana* | FJ478105 |
| 91 | *Pestalotiopsis versicolor* | AF405298 |
| 92 | *Pestalotiopsis* sp. | HQ023109 |
| 93 | *Pestalotiopsis* sp. | KR085971 |
| 94 | *Phaeosphaeria calamicola* | KY511429 |
| 95 | *Phaeosphaeria oryzae* | KM434269 |
| 96 | *Phaeosphaeria papayae* | KT224848 |
| 97 | *Phaeosphaeriopsis musae* | KR056291 |
| 98 | *Phaeosphaeriopsis* sp. | KR012892 |
| 99 | *Phoma eupyrena* | AJ890436 |
| 100 | *Phoma glomerata* | AY183371 |
| 101 | *Phoma glomerata* | EU273521 |
| 102 | *Phoma herbarum* | MF120206 |
| 103 | *Phoma leveillei* | KT963795 |
| 104 | *Phoma macrostoma* | LN714588 |
| 105 | *Phoma pomorum* | AY904062 |
| 106 | *Phoma violacea* | LN833555 |
| 107 | *Phoma* sp. | KT150649 |
| 108 | *Phomopsis liquidambari* | EU273505 |
| 109 | *Phomopsis* sp. | KX020566 |
| 110 | *Pithomyces maydicus* | HG933803 |
| 111 | *Schizophyllum commune* | KP202299 |
| 112 | *Schizophyllum fasciatum* | L43385 |
| 113 | *Stagonosporopsis cucurbitacearum* | KU059901 |
| 114 | *Stagonosporopsis ligulicola* | KJ868166 |
| 115 | *Xylaria allantoidea* | KR534657 |
| 116 | *Xylaria atrosphaerica* | KP133340 |
| 117 | *Xylaria* sp. | KC507212 |
